# COVID-19 and bank performance in dual-banking countries: an empirical analysis

**DOI:** 10.1007/s11573-022-01093-w

**Published:** 2022-05-05

**Authors:** Amal Alabbad, Andrea Schertler

**Affiliations:** 1 LaPenta School of Business, Iona College, 715 North Avenue, New Rochelle, NY 10801 USA; 2 School of Business, Economics and Social Sciences, University of Graz, Universitätsstr. 15, 8010 Graz, Austria

**Keywords:** Islamic banking, COVID-19 policy measures, Finance income, Interest income, Stock price response, PLS arrangements, G21

## Abstract

We explore how banks’ income and stock prices respond to the COVID-19 policy measures in countries with the dual-banking system, and whether Islamic banks over- or underperform compared to conventional banks. Applying two-way fixed-effect regressions, we document that the changes in Islamic banks’ finance income as well as net income decline as much during the COVID-19 pandemic as the changes in interest and net income of conventional banks. Event-study tests show that the stock prices of Islamic banks respond as negatively as the ones of conventional banks to workplace closures. We do, however, document that the two types of banks respond differently to income support schemes. The change in Islamic banks’ finance income and net income increase significantly more compared to that of their conventional peers when governments install income support initiatives. Also, Islamic banks’ stock prices respond more positively to the income support programs than the ones of conventional banks. Because we control for investment banking activities and services to large clients, our findings on the stronger response of Islamic banks to income support programs seem to result from Islamic banks’ focus on private customers who are supported during the pandemic. Overall, we conclude that the *Shariah* compliance does not limit the adverse impact of the COVID-19 crisis on Islamic banking, but that Islamic banks’ performance responds more positively to income support initiatives than the one of conventional banks.

## Introduction

The ongoing global COVID-19 crisis has led to major lockdowns across countries which have adversely affected the economic activities around the globe. The International Monetary Fund (IMF) describes the COVID-19 crisis as “a crisis like no other” (IMF 2020). Both the IMF and the Economist Intelligence Unit projected that the global economic growth is contracted by about − 4.2% in 2020, much worse than during the 2008 global financial crisis and the steepest decline since 1946. One important question is how the COVID-19 pandemic affects banks in general and their performance in particular. Banks are expected to play a key role in absorbing part of the shock since the creditworthiness of most of their borrowers declines and the collateral attached to the loans loses value as economic activity is significantly disrupted (Acharya and Steffen 2020). We have three purposes to study accounting- and market-based performance of banks residing in countries with the dual-banking system, where both Islamic and conventional banks^1^ co-exist. We examine whether the COVID-19 pandemic deteriorates the performance of Islamic banks as much as of conventional banks; whether selected economic support initiatives rebuild the performance of these two types of banks similarly, and whether particular types of Islamic banks benefit more from economic support initiatives than others.

The first purpose of this paper is to investigate whether Islamic banks’ performance suffers as much from the COVID-19 pandemic than that of conventional banks. Prior study claims that Islamic banks emerged relatively unscathed from the financial crisis of 2008 (e.g., Hasan and Dridi 2010). Islamic banks may have been more resilient to exogenous shocks because they did not take risks normal to the business of banking (Elnahass et al. 2018). We investigate net income after taxes and provisions^2^ as well as conventional banks’ interest and Islamic banks’ finance income, as these income streams might be affected the most. Interest income is an income generated from interest-yielding accounts such as loans, mortgages, and securities, and finance income is an income generated from specially designed leasing and cost-plus contracts, which we detail in the next section. Comparing the interest income of conventional banks with the finance income of Islamic banks is, however, uninformative as the levels of these income streams systematically differ.^3^ Therefore, we apply two-way fixed-effects (TWFE) regressions on the timely change in these income streams to test whether the changes in finance income we observe for Islamic banks when COVID-19 policy measures are implemented differ from the changes in interest income for their conventional peers. Using quarterly data from 2014 to 2021, we find that changes in finance/interest income for banks residing in dual-banking countries are negatively affected in the course of the pandemic and that changes in the finance income of Islamic banks are hit as severely as the changes in the interest income of conventional banks.

To examine the market-based performance of banks, we apply the dates of the first workplace closing recommendation in a traditional event study. In our test routine, we control for the fact that the COVID-19 policy measures affect all banks headquartered in a country and that many bank stocks in dual-banking countries are thinly traded. In line with the results on banks’ accounting-based performance, we find that both Islamic and conventional banks’ stock prices decline by a similar amount. The average abnormal return cumulated from ten days before to the day of the countries’ first workplace closure accounts for − 6.06%. This drop is broadly in line with the recent findings that the stock prices of banks lost relatively more value in March and April of 2020 than the ones of nonfinancial firms (Demirgüç-Kunt et al. 2021). Therefore, concerning the accounting- and market-based performance, we conclude that Islamic banks perform similarly to conventional banks in the course of the pandemic.

The second purpose is to investigate the role of economic support programs for the banks’ performance. In response to the onset of the COVID-19 pandemic, many countries have launched programs, such as income support and debt relief, as part of their stimulus packages. While income support programs can be in form of covered salaries or direct cash payments to those who lost their jobs, debt relief programs assist to alleviate some of the borrowers’ financial burden. From such programs, banks are expected to benefit because income support and debt relief schemes improve bank customers’ ability to meet their financial obligations. Do Islamic and conventional banks respond similarly to the announcement of these economic support programs? Two potential sources may initiate different responses. First, the two modes of banking may differ systematically in their degree of providing investment banking services. Banks with a strong focus on investment banking might be less affected by the pandemic, and therefore are less likely to benefit from support initiatives. Second, the customer base may systematically differ between Islamic and conventional banks, and the customers’ cash-flow sensitivity towards economic support initiatives may also vary. Our focus is on the second explanation. We generate text-based measures from the banks’ business descriptions to examine how the two types of banks differ in their business segments. We find that Islamic banks are less likely to offer investment banking services and services to large customers than conventional banks, but both banks similarly service small and medium-sized enterprises (SMEs), which received substantial support in the course of COVID in the countries we consider. In their business description, Islamic banks state more often to provide private client financings, such as private loans and residential mortgages, than conventional banks. These customers may benefit the most from income support schemes. To rule out that the result of our comparison test is driven by investment banking and large customer services, we use a propensity-score matching approach to select for each Islamic bank a conventional bank with similar characteristics. Our accounting-based performance analysis shows that the changes in finance income and net income of Islamic banks are more positively affected than the respective changes in interest income and net income of conventional banks when income support is in place. Our stock price analysis shows that the 3-days cumulative abnormal return when income support is introduced is higher for Islamic banks than for conventional banks. This result also holds when we eliminate confounding events from a propensity-score matched sample. However, we find no systematic difference in performance changes between Islamic and conventional banks when debt relief programs are implemented.

The third purpose is to investigate whether profit-and-loss-sharing (PLS)-consistent financing products in Islamic banks moderate performance changes to economic support schemes. PLS arrangements are not employed by all Islamic banks as these arrangements are claimed to suffer from severe agency problems. For this test, we hand-collect information on various Islamic income positions before the pandemic started and test whether the use of PLS arrangements moderates how banks’ performance changes with the existence of economic support schemes. Worthwhile to note is that only 50% of the Islamic banks in our sample employ PLS arrangements. Our findings reveal that the net income of Islamic banks that do not engage with PLS financing arrangements benefits more from economic support than that of PLS employing Islamic banks. We do not find signs in line with agency problems because economic support similarly fosters the finance income of both PLS and non-PLS employing Islamic banks.

This study contributes to two strands of the literature, which we discuss in the next section. The first strand of the literature investigates whether Islamic banks outperform conventional banks (e.g., Beck et al. 2013; Izzeldin et al. 2021). We contribute to this literature strand by examining whether the accounting- and market-based performance of Islamic and conventional banks changes similarly in the course of the COVID-19 pandemic. The second fast-growing strand of the literature covers the influence of COVID-19 and COVID-induced policies on banks and their behavior (e.g., Demirgüç-Kunt et al. 2021; Beck and Keil 2021). We contribute to this strand of the literature not only by focusing on an under-researched bank sample but also by investigating how economic support schemes affect the performance of banks in countries with dual-banking systems. There is also a growing body of studies on the intersection of the two previously mentioned literature strands (e.g., Hasan et al. 2021; Mirazae et al. 2021). We are not aware of any study that investigates how COVID-19 income support schemes affect banks’ accounting- and market-based performance and differentiates between Islamic and conventional banks as well as PLS and non-PLS employing Islamic banks. Differentiating between Islamic and conventional banks is insightful because the sector orientations of the two types of banks likely differ. It is also important to distinguish between Islamic banks with and without PLS contracts because PLS schemes in finance products are claimed to create significant agency problems. Our study sheds light on these differences and aims to fill this gap.

Section 2 introduces Islamic banking, discusses the related literature and how economic support schemes are related to bank performance. Section 3 presents the sample construction, introduces our control variables, and describes our propensity-score matching. Section 4 discusses our results on how the COVID-19 pandemic and economic support changes the finance/interest income and net income of Islamic and conventional banks. Section 5 delivers event-study tests on several important dates when COVID-19 policy measures are introduced. Section 6 concludes.

## Islamic banking, related literature and economic support schemes

### An introduction to Islamic banking

Islamic or *Shariah-*compliant finance refers to financial activities that adhere to the principles of *Shariah,* or the Islamic code of law. Under *Shariah* law, the main principles, on the one hand, are the prohibition of activities associated with *Riba*, which is defined as a premium or interest that is paid by the borrower to the lender, the prohibition of financing in illicit sectors such as weapons, drugs, alcohol, and pork, and the prohibition of speculative activities (*Gharar*). Therefore, derivatives are not readily accepted in Islamic finance as permissible financial instruments due to their often speculative and unfunded nature. On the other hand, there are the principles of risk-sharing and that all transactions have to be backed by a real economic transaction that involves a tangible asset. To comply with *Shariah* law, Islamic banks develop specific products, called *Shariah*-compliant financial products, which avoid interest-bearing transactions and are associated with a certain degree of risk-sharing.

The contracts that Islamic banks offer can be grouped into PLS and non-PLS contracts. With respect to the asset side of the balance sheet, PLS financing contracts are mainly *Mudaraba* and *Musharaka* contracts (see Table 1 for more details on contract definition) that involve partnership contracts and provide equity participatory finance rather than debt finance. *Mudaraba* is a form of business partnership between the bank and borrowers, or entrepreneurs, which is based strictly on a PLS scheme where profits are shared at a predetermined ratio while losses are borne exclusively by the bank with limited liability provisions covered for the entrepreneur. Therefore, in the event of losses, the entrepreneur makes no financial contributions and has no liability for losses and s/he receives no remuneration. Although the entrepreneur has the ultimate control over the business, major investment decisions have to be approved by the bank. Similar to the *Mudaraba* arrangement is the *Musharaka* contract where the Islamic bank is one of several investors with profits and losses being shared among all investors in proportion to their participation. Looking at the liability side, PLS consistent funding comes from *Mudaraba*-based contracts in which depositors’ funds are pooled. Such investment deposits can be either linked to the bank’s overall profit level or a specific investment account on the asset side of the bank’s balance sheet. Therefore, “depositors” or investment account holders have payoffs that resemble more closely the payoffs of equity holders of conventional banks, who earn dividends for their investment (Khan 1991), than the payoffs of creditors of those conventional banks.^4^

**Table 1:** Basic terminology of Islamic banking

Term	Explanation
Profit-loss sharing (PLS) contracts	
*Mudaraba* (Trustee finance contract)	It is a partnership contract as *rabb-ul-mal* (financer or depositor) provides the entire capital needed to finance a project while the entrepreneur or the bank offers his/her labor and expertise. Profits are shared between them at a certain fixed ratio, whereas financial losses are exclusively borne by *rabb*-*ul*-*mal* unless there is negligence from the side of the entrepreneur. The liability of the entrepreneur is limited only to his/her time and effort
*Musharaka* (Equity participation)	The bank enters an equity partnership agreement with one or more partners to jointly finance an investment project. Profits (and losses) are shared in relation to the respective capital contributions
Non-PLS contracts	
*Ijara* (Lease, lease purchase)	A client leases a particular product, a vehicle or equipment, for a specific sum and a specific time. This contract takes the form of an operating lease with an option to renew the contract each time the lease terminates. In the case of lease purchase, each payment includes a portion that goes toward the final purchase and transfer of ownership of the product
*Istisna* (Deferred payment, deferred delivery)	A manufacturer (contractor) agrees to produce (build) and to deliver a certain good (or premise) at a given price on a given date in the future. The price does not have to be paid in advance (in contrast to buying *Salam*, see below). It may be paid in installments or part may be paid in advance with the balance to be paid later, based on the preference of the parties
*Murabaha* (Mark–up financing)	It is a cost-plus sale as the bank purchases the good or commodity for a fixed cost and then sells it to the client at a higher price, which is referred to as the markup. The sale price is predetermined and is specified in the contract between the client and the Islamic bank while the purchase price is negotiated between the supplier and the banks
*Salam* (Pre-payment, deferred delivery)	A client or a wholesaler makes an advance payment to the Islamic banks to finance a product or good to be delivered to the client in the future. In turn, Islamic bank makes an advance payment to a supplier for the same good to be received by the bank on a future date

Source: Errico and Farrahbaksh (1998) and El-Hawary et al. (2004)

Aggarwal and Yousef (2000) argue that Islamic banks face severe agency problems in their attempts to provide funds to entrepreneurs based on PLS arrangements. Looking closely at the PLS contracts, *Musharaka* financing encounters moral hazard problems associated with *ex-post* information asymmetry. The entrepreneur (borrower), for example, has an incentive to under-declare or artificially reduce reported profit (Mills and Presley 1999). Besides, in the case of *Mudaraba* contract, the entrepreneur has an incentive to undertake high-risk projects because s/he is given a call option whereby s/he gains on the upside but bears no losses at all on the downside. Also, in *Mudaraba* financing, the bank bears all the risk associated with the capital, but the management and control of the project are mostly in the hands of the entrepreneur. This think accentuates the principal-agent problems associated with PLS financing (Dar and Presley 2000).

As a result, Islamic banks deviate from the PLS financing mode (Aggarwal and Yousef 2000; Ibrahim 2006; Matoussi and Grassa 2010; Grassa 2012). Several studies document a low percentage of PLS financing mode and explain this deviation from the PLS financing scheme by the fact that these financing products are riskier and require more costly monitoring (Matoussi and Grassa 2010; Grassa 2012), while others indicate that non-PLS financing contracts may satisfy the need of the majority customer of the bank more than PLS financing contracts (Ibrahim 2006). However, Othman et al. (2017) report that banks that offer a huge amount of PLS financing tend to be more efficient than other Islamic banks except for the 2008 financial crisis. Due to the agency problems associated with the PLS financing contracts, Islamic banks tend to engage more with assets-based-financing instruments that do not follow the PLS scheme. These instruments consist of *Murabaha,* or cost-plus contract, which is one of the most applied forms of Islamic financial contracts where the financier, or the bank, buys assets and then sells those assets to the client at a higher price. *Ijara* is a form of leasing contract whereby a legal title of a leased asset is transferred to the lessee after the expiry of the leasing period*.* Other non-PLS contracts include *Istisna* which is a contract to deliver assets from manufacturer to client for installment payment or on delivery and *Salam* which is another form of a forward sale contract whereby the payment of goods or commodities is paid in advance and the delivery takes place on a specified date in the future.

Islamic banks’ governance and management processes must also abide by Islamic law. One of the key features that distinguishes the governance structure of Islamic banks from that of their conventional counterparts is the institution of the *Shariah* Supervisory Board. In addition to the regular board of directors and routine executive and other operational committees, the *Shariah* Supervisory Board acts as an independent control mechanism to certify that all financial contracts, transactions, and further activities of the bank are compliant with *Shariah* law. This board works as an additional layer of monitoring and oversight as well as a constraint on the operation as it might restrain the board of directors and management from engaging in aggressive risk-taking activities (Mollah and Zaman 2015). Therefore, one unique type of risk Islamic banks face is the *Shariah* compliance risk due to the bank’s compliance with *Shariah* law (Khan and Ahmed 2001).

### Related literature

The first strand of the literature relevant to our study tackles the debate on whether Islamic banks outperform conventional banks. Early work has applied various metrics to measure relative performance, such as profitability (Samad 2004; Rashwan 2010), liquidity (Hassan and Bashir 2003; Beck et al. 2013), and efficiency (Bader et al. 2008; Abdul-Majid et al. 2010; Beck et al. 2013; Izzeldin et al. 2021; Safiullah and Shamsuddin 2019) to examine whether Islamic banks are better or worse than their conventional peers, but the findings are mixed. Research also examines whether Islamic banks are more or less stable than conventional banks [we refer the reader to the extensive literature review by Ghassan and Krichene (2017)]. Several studies point out that size matters much in the debate of whether Islamic banks are more stable than conventional peers (e.g., Čihák and Hesse 2010; Abedifar et al. 2013; Alqahtani and Mayes 2018). Overall findings supported the claim that Islamic banks are more resilient to financial shocks compared to conventional banks (Hasan and Dridi 2010; Perry and Rehman 2011; Beck et al. 2013; Mollah et al. 2017). Perry and Rehman (2011) highlight that “while many of the conventional banks suffered major loses in the aftermath of the sub-prime mortgage crisis, most banks following the Islamic system were largely profitable”. Hasan and Dridi (2010) point out that Islamic banks did not announce substantial write-offs and had higher asset growth than conventional banks during the global financial crisis. Applying alternative stability approaches, recent studies however report mixed results (Doumpos et al. 2017; Vasileios et al. 2017).

The second fast-growing strand of the literature covers the influence of COVID-19 on banks and their behavior. Although it is a health crisis, the recent outbreak of the COVID-19 pandemic has created unprecedented panic and uncertainty over global business activities and has driven the global economy toward depression. Scholars examine how banks have been affected by the pandemic. Recent research investigates stock price responses of banks in industrialized and developing countries (Demirgüç-Kunt et al. 2021), loan spreads of syndicated loans (Hasan et al. 2020), lending growth (Greenwald et al. 2020; Beck and Keil 2021), increased lending through credit line drawdowns (Acharya and Steffen 2020; Chodorow-Reich et al. 2020), how depositors respond to the COVID-19 crisis (Levine et al. 2020), how banks accommodate their customers’ liquidity demand (Li et al. 2020), and how this pandemic impacts global banking stability (Elnahass et al. 2021). For instance, Elnahass et al. (2021) provide evidence that, in the global banking sector, the COVID-19 outbreak has detrimental effects on accounting- and market-based performance and financial stability.

There is also a growing body of studies on the intersection of the two previously mentioned literature strands. Elnahass et al. (2021) find that Islamic banks have significantly higher asset risk, with a marginally higher insolvency risk, but exhibit a marginally higher return on assets than their conventional counterparts. Yet, their overall findings indicate that Islamic banks suffer from the COVID-19 pandemic, just as conventional banks do. Mirazae et al. (2021) report that the stock returns of Islamic banks are about 10–13% higher than those of conventional banks during the initial phase of the COVID-19 crisis and claim that the higher level of Islamic banks’ pre-crisis efficiency would explain the behavior of stock returns. Hasan et al. (2021) find that Islamic and conventional stock price indices strongly co-move from January to November of 2020. Rizwan et al. (2021) document an increase in banks’ systemic risk during the first half, followed by a recovery in the second half of 2020. They show that Islamic banks while earning abnormal returns, pose significantly less spillover to others relative to conventional banks. Focusing on Islamic banks only, Mansour et al. (2021) find that Islamic banks’ responses in profitability, non-performing financing, size, and stability to the pandemic are not uniform across countries.

### Economic support schemes and bank performance

In response to the onset of the COVID-19 pandemic, many countries have launched income support and debt relief programs as part of their stimulus packages to support households and businesses. An income support program can be in the form of covered salaries or direct cash payments to those who lost their jobs, including payments to their firms to cover payroll. For instance, the stimulus packages amount to 1.2% of Gross Domestic Product (GDP) for Egypt and 4.2% of GDP for Bahrain to support the most vulnerable households with social assistance programs, and targeted cash transfers provided to workers (OECD 2020). Saudi Arabia’s government compensated 60% of employees’ salaries for three months to prevent companies from laying off employees, under a scheme covering up to 70% of Saudi Arabia’s workers in the most affected companies (OECD 2020). Debt relief programs can be in form of deferring loan repayment, restructuring loans without charges, and offering long-term loans for companies as assistance to alleviate some of their financial burdens. Under Saudi Arabia’s program, for example, SAR 30 billion was allocated for banks and financing companies to delay loan payments due from SMEs for six months. The package provides SAR 13.2 billion to SMEs through bank loans to help with their operations. Also, a SAR 6 billion loan guarantee program was granted to SMEs as a relief from their finance costs. Other countries such as United Arab Emirates (UAE), Kuwait, Jordan, and Tunisia established stimulus package that includes allowing loan repayment deferrals, lowering banks’ reserve requirements, and providing zero-interest-rate collateralized loans to banks to support SMEs and/or expand the coverage of guarantees on SME loans (OECD 2020).

We postulate that economic support schemes can be a significant moderator of banks’ performance because they improve bank customers’ ability to meet their financial obligations. Income support schemes increase the chances that private borrowers can repay their personal loans and residential mortgages. Indirectly, income support may also increase the ability of corporate borrowers to meet their financial duties as such support could reduce the costs of the companies. Debt relief schemes may be more relevant for companies’ ability to meet their commitments as these schemes would not protect workers from being laid off or against reduced work time. We are aware of only one study focusing on the effects of debt relief programs on bank stock performance. Demirgüç-Kunt et al. (2021) document that borrower assistance programs moderate the adverse impact of the crisis. They find that banks headquartered in developed countries show large positive abnormal returns following the announcement of these programs, while banks in developing countries do not show positive abnormal returns most likely because there is less room for fiscal expansion in these countries. We do not only investigate stock performance and net income but also the finance income of Islamic banks and the interest income of conventional banks as we expect that these income streams respond more to income support and debt relief programs than non-finance/non-interest income. The reason for this is that non-finance/non-interest income contains income from other banking services, such as investment banking services, which are less affected by the pandemic.

Do Islamic and conventional banks respond differently to economic support schemes? The answer to this question depends on the banks’ customer structure. Banks with many private customers may profit more from income support schemes than banks with many corporate customers. Moreover, banks with many SMEs in their customer base may benefit more than banks with large customers because economic support is focused on helping SMEs in these countries. Thus, the asset structure of Islamic vs. conventional banks helps determine which type of bank benefits more from the economic support programs. Unfortunately, the sectoral allocation of the asset portfolio is rarely known for banks in dual-banking countries. The exceptions are as follows: In Bahrain as reported in Farooq et al. (2018), the percentage of personal/consumer finance of Islamic banks cater to 23.6% vis-à-vis a lower 13.7% offered by conventional banks. The two types of retail banks also differ with respect to residential mortgages (10.3% versus 7.2%). For wholesale banks, these differences are even more pronounced. Islamic (conventional) banks have 21.3% (3.4%) in personal/consumer finance and 10.9% (0.3%) in residential mortgages. In Turkey, SME lending of Islamic banks accounts for about 46% of the total lending portfolio compared to 25% lending from conventional banks (World Bank–IsDB 2015). In Saudi Arabia, a report indicates that up to 90% of SMEs are only seeking *Shariah*-compliant banking services and avoiding conventional financing banking (IFC 2014). Research also reports that billions of funds for SME financing in Malaysia are disbursed by Islamic banks via various types of financing (see, for example, Kamel 2019), and more than about 41% of customers for Islamic banks in Indonesia are households, while only 6.9% of their clients are financial intermediaries (Thaker et al. 2020). Thus, if Islamic banks earn most finance income either from private customers or SMEs, we would expect that income support has a stronger positive effect on the finance income of Islamic banks than on the interest income of conventional banks.

## Sample, controls and matching

To construct our sample, we follow Beck et al. (2013) and focus only on countries with both Islamic and conventional banks.^5^ For these dual-banking countries, we collect information on all banks that have been publicly listed and are included in Refinitiv. Table 2 shows that we start with 331 banks headquartered in 23 countries. These banks are either classified as banks, consumer lenders, or investment banks according to the sector classification of Refinitiv. To distinguish between Islamic and conventional banks, we hand-collect information from the banks’ annual reports and homepages. Among the 331 banks, we identify 52 banks operating under *Shariah* law.

**Table 2:** Banks and countries

Country	ALL	Islamic banks	With income information		With stock prices		First enaction of …		
			ALL	Islamic banks	ALL	Islamic banks	Workplace closure	Income support	Debt relief
Bahrain	16	6	10	5	3	1	18-Mar-20	01-Apr-20	01-Feb-20
Bangladesh	30	7	29	6	29	6	19-Mar-20	13-Apr-20	19-Mar-20
Cayman	1	0					23-Mar-20		
Egypt	14	3	16	3	10	3	16-Mar-20	13-Apr-20	22-Mar-20
Gambia	1	0	1	0			27-Mar-20	09-Oct-20	08-Jun-21
Indonesia	36	1	33	1	24	0	15-Mar-20	04-Sep-20	01-Apr-20
Iraq	21	3	21	3			17-Mar-20	17-May-20	17-May-20
Jordan	15	2	15	2	7	1	18-Mar-20	29-Mar-20	18-Mar-20
Kuwait	26	8	10	5	21	7	11-Mar-20	01-Apr-20	07-May-20
Lebanon	6	0	6	0	1	0	06-Mar-20	09-Sep-21	08-Apr-20
Malaysia	18	0	11	0	15	0	18-Mar-20	06-Apr-20	01-Apr-20
Pakistan	32	5	21	2	26	4	23-Mar-20	09-Apr-20	16-Apr-20
Palestine	3	0	2	0			06-Mar-20		21-Apr-20
Qatar	8	3	8	3	7	3	17-Mar-20	28-Mar-20	24-Jul-20
Saudi Arabia	12	4	12	4	11	4	16-Mar-20	03-Apr-20	11-Apr-20
Senegal	1	0					16-Aug-20	03-Apr-20	01-Apr-20
Singapore	9	0	6	0	7	0	07-Apr-20	01-Apr-20	20-Apr-20
Sudan	1	1	1	1			14-Mar-20	15-Apr-20	
Syria	11	1	11	1			13-Mar-20		
Thailand	19	0	9	0	16	0	17-Mar-20	01-Apr-20	01-Apr-20
Tunisia	11	0			9	0	18-Mar-20	21-Mar-20	21-Mar-20
Turkey	17	2	14	1	15	1	16-Mar-20	07-Apr-20	12-Apr-20
UAE	23	6	22	6	9	5	01-Mar-20		01-Apr-20
Total	331	52	258	43	210	35			

The number of publicly listed banks and Islamic banks located in dual-banking countries is shown. The remainder is conventional banks. The numbers are shown for all publicly listed banks in dual-banking countries, for banks with income information, and banks with stock price information available. The dates of the workplace closure, income support, and debt relief (Hale et al. 2020) are also reported

**Fig. 1:**
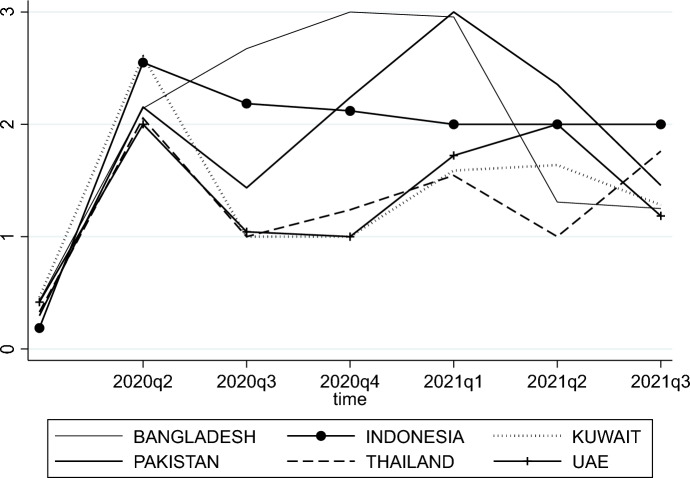
This figure presents the quarterly average of the daily reported workplace closure indicator from Hale et al. (2020) for selected countries

To measure the start of COVID-19 in dual-banking countries, we use an indicator called workplace closures from the ‘*Variation in government responses to COVID-19*’ report prepared by the Blavatnik School of Government at the University of Oxford (see, Hale et al. 2020). Workplace closures belong to the policy actions governments have taken in attempts to contain the severity of this pandemic. This indicator is reported daily and can take four different values. If the indicator is 0, no measures are in effect. If it is 1, a closing is recommended; if the indicator equals 2 [3] a required closing or work from home is in place for some sectors or categories of workers [all-but-essential workplaces, e.g., grocery stores, doctors]. In Fig. 1, we depict the quarterly averaged workplace closure indicator for selected countries to show that (i) the first workplace closure requirement takes place at the same point in time, and (ii) the severity of lockdown requirements differs only slightly over time across the countries selected. For our event study, we define the day of the first occurrence of a recommended or required closing (indicator differs from zero for the first time) as our workplace closure event. For most countries in our sample, the first recommended or required workplace closure takes effect in the first quarter of 2020 (Table 2).

We consider two economic support schemes that countries around the globe implemented that are positive events for banks because these actions may help bank customers to meet their obligations. The income support scheme is our first event type. The indicator reported by Hale et al. (2020) equals 1 if less than 50% of lost salary are replaced and 2 when the government replaces 50% or more of lost salary. Many but not all countries in our sample install income support programs (Table 2). Regarding debt relief schemes, Hale et al. (2020) offer an indicator that measures whether the government is freezing a few or many types of financial obligations. Almost all countries in our sample install debt relief schemes, the only exceptions are Cayman, Sudan, and Syria (Table 2).

In Table 2, we also depict the number of banks that we have available when investigating income and stock price responses. Banks’ income is considered from Q3 2014 to Q3 2021; the third quarter of 2014 is the first quarter when income information for Islamic banks becomes available in Refinitiv. In our income analysis, we have a maximum of 258 banks, 43 of which are Islamic banks. When we study stock price responses, we consider 210 banks, 35 of which are Islamic banks. The lower number of banks in our stock price analysis is because stock index information is missing and/or because banks’ stock prices are constant through time. More specifically, we remove banks from the abnormal return analysis, if more than 50% of their daily return observations in 2019/2020 are equal to zero. These stocks are either not traded or they have been delisted in the meantime.^6^

We present descriptive statistics for the COVID-19 measures and control variables in Panel a of Table 3, their correlations in Panel b, and their definitions and sources in an Appendix. To describe relevant dimensions in the business models of the banks, we use the business description from Refinitiv and apply various wording lists (see the Appendix for all words considered) to generate text-based variables. The first list contains investment banking, M&A, mergers, corporate advisory to generate a binary indicator on whether or not the bank provides investment banking services (D_*INVESTMENT*). Banks offering investment banking services might be less exposed to the COVID pandemic. The second list contains large borrowers/ large customers/ large clients as these services often have an international investment and financing focus. If the bank has an eye on large customers and clients, the indicator D_*LARGE* equals one, and zero otherwise. The third word list captures whether the bank explicitly states to serve small customers. The final word list captures whether the bank explicitly states to serve private clients and to offer residential mortgages. The mean values of these indicators (see Panel a) show that conventional banks state more often to offer investment banking services and serve larger as well as smaller clients than Islamic banks while Islamic banks state more often to finance private customers than conventional banks. Noteworthy, 23.1% of conventional banks explicitly state that they offer private customers and household finance compared to 44.2% of the Islamic banks.

**Table 3:** Characteristics of Islamic and conventional banks

Panel a: Independent variables					
	Islamic banks		Conventional banks		Equality test
	Mean	SD	Mean	SD	
LOCK	1.557	0.724	1.654	0.760	0.049
InSu	0.888	0.796	0.800	0.704	0.061
DeRe	1.315	0.739	1.276	0.764	0.437
D_INVESTMENT	0.234		0.353		0.000
D_LARGE	0.001		0.084		0.000
D_SMALL	0.257		0.369		0.000
D_PRIVATE	0.439		0.227		0.000
VaR	0.017	0.009	0.017	0.011	0.291
SD	0.018	0.008	0.019	0.010	0.000
Z	24.251	18.637	24.213	18.417	0.953
Tier 1	16.872	7.268	15.278	5.405	0.000
EQ	0.148	0.128	0.147	0.113	0.789
Lt debt	0.009	0.020	0.044	0.053	0.000
ROA	0.012	0.013	0.012	0.014	0.749
TA	1.28E+10	1.81E+10	2.48E+10	5.11E+10	0.000

Panel b: Correlations															
		(1)	(2)	(3)	(4)	(5)	(6)	(7)	(8)	(9)	(10)	(11)	(12)	(13)	(14)
LOCK	(1)	1													
InSu	(2)	0.682	1												
DeRe	(3)	0.859	0.649	1											
D_INVESTMENT	(4)	− 0.013	0.042	0.042	1										
D_LARGE	(5)	− 0.023	− 0.004	− 0.007	0.137	1									
D_SMALL	(6)	0.034	− 0.001	0.055	0.041	− 0.199	1								
D_PRIVATE	(7)	− 0.008	− 0.001	0.021	0.088	0.027	− 0.111	1							
VaR	(8)	− 0.006	0.046	− 0.011	0.084	− 0.071	0.082	− 0.017	1						
SD	(9)	0.060	0.037	− 0.004	− 0.114	− 0.026	− 0.015	0.002	0.490	1					
log(Z)	(10)	− 0.034	0.056	− 0.018	0.196	0.031	0.013	− 0.027	− 0.074	− 0.255	1				
Tier 1	(11)	0.049	0.089	0.004	− 0.121	− 0.002	− 0.216	0.057	− 0.211	0.052	0.104	1			
EQ	(12)	− 0.012	− 0.015	− 0.088	− 0.163	− 0.062	− 0.240	0.011	0.105	0.183	− 0.025	0.785	1		
Lt debt	(13)	0.051	0.055	0.078	0.109	0.039	0.220	− 0.085	0.238	0.086	0.015	− 0.292	− 0.247	1	
ROA	(14)	− 0.096	− 0.040	− 0.082	0.078	0.100	− 0.035	0.046	− 0.011	− 0.080	0.120	0.049	0.059	− 0.026	1
Log(TA)	(15)	0.024	0.138	0.101	0.336	0.089	0.143	− 0.008	− 0.031	− 0.253	0.486	− 0.225	− 0.438	0.242	0.107

Panel c: Propensity score					
	(1)	(2)	(3)	(4)	(5)
VaR (y− 1)	2.972*	2.633	2.859*	2.523*	2.927**
	(1.720)	(1.617)	(1.496)	(1.365)	(1.443)
log(Z (y− 1))	0.019	0.046	0.045	0.052	0.049
	(0.053)	(0.056)	(0.055)	(0.052)	(0.054)
EQ (y− 1)	− 0.214	− 0.568**	− 0.407**	− 0.447**	− 0.479**
	(0.221)	(0.223)	(0.206)	(0.192)	(0.200)
Lt debt (y− 1)	− 1.965***	− 2.015***	− 2.059***	− 2.018***	− 2.033***
	(0.368)	(0.394)	(0.390)	(0.363)	(0.377)
ROA (y− 1)	− 0.520	0.118	− 0.301	− 0.135	− 0.163
	(0.711)	(0.588)	(0.615)	(0.591)	(0.591)
log(TA (y− 1))	0.010	− 0.031*	0.760***	0.527**	0.615**
	(0.015)	(0.018)	(0.278)	(0.247)	(0.261)
log(TA (y− 1))^2^			− 0.017***	− 0.012**	− 0.014**
			(0.006)	(0.005)	(0.006)
muslim%	0.001*				
	(0.001)				
D_INVESTMENT				− 0.116**	− 0.114**
				(0.050)	(0.054)
D_LARGE				− 0.167***	− 0.183***
				(0.055)	(0.051)
D_SMALL				− 0.056	
				(0.049)	
D_PRIVATE				0.125**	
				(0.055)	
Year FE	Yes	Yes	Yes	Yes	Yes
Country FE	No	Yes	Yes	Yes	Yes
# of obs	1490	1490	1490	1490	1490
# of banks	258	258	258	258	258
adj R^2^	0.081	0.225	0.240	0.290	0.268

Panel a reports the mean and standard deviation (SD) of our independent variables. *LOCK*, *InSu*, and *DeRe* are summarized for the years 2020 and 2021. *D_INVESTMENT*, *D_LARGE*, *D_SMALL*, and *D_PRIVATE* come from February 2022, and all other measures come from the pre-pandemic years 2014–2019. For further variable definitions, see Appendix. Panel b reports their correlations based on data from Q3 2014 to Q3 2021. In Panel c, we report results from a linear probability model on being a bank operated under *Shariah* law, which we use in later analysis to find for each Islamic bank a similar conventional bank. Annual data are used in these regressions from 2014 to 2019. Standard errors are in parenthesis and are clustered at the bank level. *, **, and *** denote statistical significance at the 10%, 5%, and 1% levels, respectively

As risk measures, we calculate the standard deviation (*SD*) of stock returns and value-at-risk (*VaR*) which we model as the 10% worst value of the annual stock return distribution. Equality tests show that the return standard deviation before the COVID-19 pandemic started is significantly lower for Islamic banks than for conventional banks, but the two banking types do not differ with respect to the value-at-risk. As these two measures are correlated (see Panel b), we only use the value-at-risk measure in our analysis. We also consider information from banks’ balance sheets and profit and loss statements. Unfortunately, several traditional risk measures such as risk-weighted assets or nonperforming loans are hardly available for the banks in our sample. However, we follow recent research (e.g., Beck et al. 2013) and calculate a z-score. The lower the z-score (*Z*), the closer is the bank to its default point. Equality tests show that the two banking types do not differ with respect to the z-score. If anything, we can conclude that Islamic banks have no higher risk than their conventional peers before the COVID-pandemic started. We further consider three financing ratios, namely a Tier 1 capital ratio, an equity ratio, and a long-term debt ratio. Islamic banks have higher Tier 1 capital ratios (*Tier* 1), lower long-term debt ratios^7^ (*Lt debt*), and similar equity ratios (*EQ*) than conventional banks. We employ the equity ratio, not the Tier 1 capital ratio in our empirical analysis because the two variables are highly correlated (see Panel b) and the number of observations available is higher for the former than for the latter. Return on assets (*ROA*) is our performance measure and an equality test shows that the two banking types do not differ in this respect. We also control for size by including the banks’ total assets (*TA*). Islamic banks are significantly smaller than their conventional peers.

One difficulty with every performance comparison of Islamic and conventional banks is that these banks may systematically differ from each other and that these differences limit the insights gained from a comparison. At least two sources of heterogeneity matter in our sample. First, we include several countries where no Islamic bank is publicly listed. Second, even within the same country, Islamic banks may fundamentally differ from conventional banks along several lines and therefore their performance may respond differently to economic support schemes. For instance, conventional banks are larger, on average, and they are more likely to do investment banking businesses than Islamic banks. Larger conventional banks systematically employ more derivatives instruments (Memmel and Schertler 2012) than smaller conventional banks. Moreover, particular economic support programs, such as borrower assistance programs, affect the performance of large banks more than that of small banks (Demirgüç-Kunt et al. 2021). Ignoring these sources of heterogeneity could cause systematic performance differences between Islamic and conventional banks. Therefore, we apply a matching approach based on propensity scores to identify for each Islamic bank a conventional bank with similar characteristics.

We use a linear probability model where the dependent variable is a dummy variable that equals one if the bank operates under *Shariah* law, and zero if it is a conventional bank and we report results from alternative specifications in Panel c of Table 3. In column (1), we include the percentage of the Muslim population in a country and find that a bank operating under *Shariah* law is more likely in countries with a higher Muslim population. In column (2), we replace the Muslim population with a full set of country dummy variables and observe that the adjusted R^2^ increases from 8.1% in column (1) to 22.5% in column (2). The increase in adjusted R^2^ indicates that substantial country differences exist. In column (3), we shed light on a potential non-linearity in total assets by adding a squared term of total assets. While the coefficient on total assets is insignificant in column (1), total assets and total assets squared are highly significant in column (3). They indicate that the likelihood of being a bank operating under *Shariah* law first increases in total assets and decreases after a specific size of total assets is reached. Other control variables also play a role. Higher value-at-risk (*VaR*), lower equity (*EQ*), and long-term debt ratios (*Lt debt*) make having an Islamic bank more likely. As far as we can see from the data, the two modes of banks do not differ with respect to their overall risk measured by z-score (*Z*).

In column (4), we add all text-based variables and find that the likelihood of being an Islamic bank is lower when investment banking services and large customer services are provided. While Islamic and conventional banks are similar with respect to financing SMEs, stating financing private clients in the business description more likely comes with a bank operating under *Shariah* law. This finding supports our argument that stronger effects of income support initiatives can be expected for Islamic banks’ income because their asset portfolio is more concentrated on financing private clients compared to that of conventional banks. The adjusted R^2^ increases from 24% in column (3) to 26.8% in column (4) with the text-based variables.

In column (5), we present the specification from which we calculate propensity scores. For each Islamic bank, we select one conventional bank whose propensity score is closest to one of the Islamic banks. The mean difference in propensity scores between the Islamic and conventional banks is as low as 3.1%. The specification does not control private client services and SME financing statements because this would eliminate the asset allocation difference between Islamic and conventional banks we are interested in. We control for investment banking and large client services, as these services are expected to influence the relationship between economic support and bank performance. A cautionary note to be considered here is that our text-based indicators have two shortcomings: They do not vary over time and they might be imperfectly correlated with the true unknown exposure of a bank in the respective business segment. However, because we lack other sources, they are the best way for us to control for investment banking and large client services, which might otherwise determine banks’ income response to the income support schemes.

## Change in income

In this section, we discuss how banks’ income changes with the COVID-19 policy measures. We first introduce the two-way fixed-effects (TWFE) regression framework which we use to analyze various income positions and afterward discuss our results.

### Income model

We test whether banks’ income declines significantly in the course of the COVID-19 pandemic and whether Islamic banks’ income declines less or more strongly during the pandemic than that of conventional banks.^8^ Our panel consists of Islamic and conventional banks from Q3 2014 to Q3 2021. To estimate this panel, we use the TWFE (entity and time) regression framework. To model the course of the pandemic, we start with the workplace closure indicator. However, it turns out that this indicator creates a variance inflation factor of more than 12 with the time-fixed effects. This multicollinearity is not surprising because workplace closures occur only in a few quarters (see, Table 2). Therefore, the effects of the pandemic are determined solely by the time-fixed effects. Our model looks like this:

1 $$\begin{aligned}{CH\_Y}_{it}&=\alpha +\delta \times {D\_IB}_{i}+{\beta }_{1}\times {VaR}_{it-1} +{\beta }_{2}\times {\mathrm{log}(Z}_{it-1})\\ &\quad+{\beta }_{3}\times {EQ}_{it-1} +{\beta }_{4}\times {Lt debt}_{it-1}+{\beta }_{5}\times {\mathrm{log}(TA}_{it-1})\\ & \quad+{\beta }_{6}\times {ROA}_{it-1}+{\mu }_{i}+{\lambda }_{t}+{\varepsilon }_{it}\end{aligned}$$

where ${Y}_{it}$ is either finance/interest income, non-finance/non-interest income, or the net income of bank *i* in quarter *t.* We consider finance/interest income because we expect that this income stream is affected the most; for comparison purposes, we also consider non-finance/non-interest income. $CH$ denotes the quarterly change calculated as ${CH\_Y}_{it}=\frac{{Y}_{it}-{Y}_{it-3}}{{TA}_{it-1}}$. We scale the quarterly change in an income position by total assets as income can be negative.^9^*D_IB* is an indicator that equals one if a bank is Islamic and zero if it is a conventional bank. All control variables are lagged by one quarter. ${\mu }_{i}$ is the bank-specific fixed effect which subsumes the effect of *D_IB* and ${\lambda }_{t}$ is the time-specific fixed effect. ${\varepsilon }_{it}$ denotes the error term. We model heteroscedasticity-consistent standard errors, which we cluster at the bank level.

To study economic support measures, we extend our model. As the income support measure is highly correlated with the debt relief measure (see, Panel b of Table 3), we include them separately. This extended model looks as follows

2 $$\begin{aligned}{CH\_Y}_{it}& =\alpha +{\delta }_{1}\times {ESI}_{it}+{\delta }_{2}\times {D\_IB}_{i}\times {ESI}_{it}+{\beta }_{1}\times {VaR}_{it-1}\\ & \quad+{\beta }_{2}\times {\mathrm{log}(Z}_{it-1}) +{\beta }_{3}\times {EQ}_{it-1}+{\beta }_{4}\times {LT debt}_{it-1}\\& \quad+{\beta }_{5}\times {\mathrm{log}(TA}_{it-1}) +{\beta }_{6}\times {ROA}_{it-1}+{\mu }_{i}+{\lambda }_{t}+{\varepsilon }_{it}\end{aligned}$$

where ${ESI}_{it}$ denotes the quarterly average of the economic support indicator*.*

### Results from various income positions

**Fig. 2:**
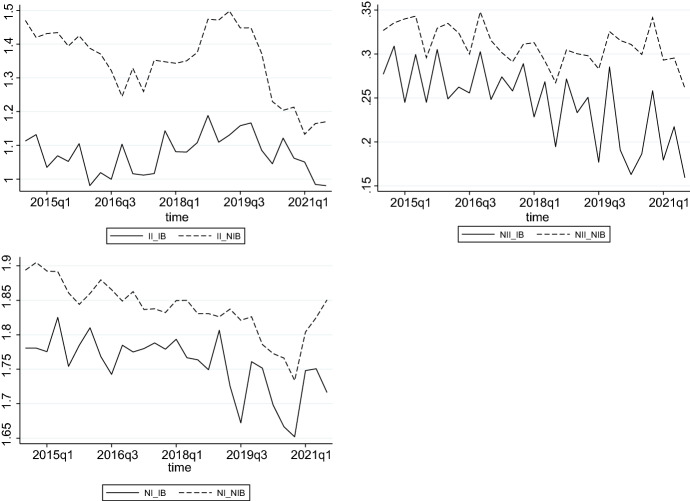
This figure presents Islamic and conventional banks’ income from finance/interest income (*II*), non-finance/non-interest income (*NII*), and net income after taxes and provisions (*NI*) in terms of total assets over Q3 2014 to Q3 2021. *_IB* refers to Islamic banks and *_NIB* refers to conventional banks

In Fig. 2, we depict averages of our income measures relative to total assets over the full sample period. The figure shows that the finance income of Islamic banks strongly comoves with the interest income of conventional banks before the start of the pandemic but on different scales. The finance income of Islamic banks is lower than the interest income of conventional banks. The non-finance income of Islamic banks also comoves with the non-interest income of conventional banks. The net income after taxes and provisions shows some differences; it fluctuates more for Islamic banks than for conventional banks.

Columns (1) to (3) of Table 4, Panel a, present the results for the change in the three income positions for the baseline model (Eq. (1)). We only present the results from relevant quarters in 2020/21 to determine the effects of the pandemic on bank income. The base group in our estimation is Q4/2019. During all COVID-quarters, we see negative changes in finance/interest income (column (1)). Non-finance/non-interest income (column (2)) as well as net income (column (3)) show positive changes in several quarters. Thus, banks in dual-banking countries had to manage decreases in interest/finance income during the COVID-19 crisis, which seems to be overcompensated by higher non-finance/non-interest income.

**Table 4:** TWFE income regressions

Panel a: Quarters									
	(1)	(2)	(3)	(4)	(5)	(6)	(7)	(8)	(9)
	All banks			All banks			Matched banks		
	CH_II	CH_NII	CH_NI	CH_II	CH_NII	CH_NI	CH_II	CH_NII	CH_NI
Q1/2020	− 0.175***	0.287***	0.411***	− 0.179***	0.340***	0.446***	− 0.215***	0.209	0.333*
	(0.025)	(0.089)	(0.088)	(0.022)	(0.104)	(0.102)	(0.053)	(0.172)	(0.182)
Q2/2020	− 0.359***	0.208***	0.348***	− 0.383***	0.257***	0.381***	− 0.469***	0.195	0.196
	(0.034)	(0.077)	(0.092)	(0.036)	(0.090)	(0.107)	(0.062)	(0.179)	(0.174)
Q3/2020	− 0.471***	− 0.066	0.183**	− 0.492***	− 0.069	0.156*	− 0.591***	0.058	0.176**
	(0.044)	(0.049)	(0.076)	(0.042)	(0.052)	(0.081)	(0.106)	(0.071)	(0.074)
Q4/2020	− 0.305***	− 0.123	− 0.247**	− 0.310***	− 0.169	− 0.303**	− 0.372***	− 0.045	− 0.036
	(0.035)	(0.086)	(0.105)	(0.040)	(0.103)	(0.127)	(0.061)	(0.135)	(0.158)
Q1/2021	− 0.210***	− 0.166*	− 0.050	− 0.183***	− 0.245**	− 0.124	− 0.240***	− 0.101	0.122
	(0.035)	(0.085)	(0.111)	(0.036)	(0.095)	(0.131)	(0.076)	(0.139)	(0.178)
Q2/2021	− 0.146***	0.152*	0.361***	− 0.107***	0.187**	0.380***	− 0.168**	− 0.024	0.146***
	(0.032)	(0.084)	(0.085)	(0.032)	(0.088)	(0.087)	(0.069)	(0.044)	(0.055)
Q3/2021	− 0.195***	− 0.174***	0.184***	− 0.195***	− 0.157***	0.184**	− 0.284***	− 0.097	0.135**
	(0.041)	(0.055)	(0.065)	(0.034)	(0.046)	(0.072)	(0.097)	(0.063)	(0.062)
D_IB × Q1/2020				0.027	− 0.319**	− 0.216	0.068	− 0.174	− 0.090
				(0.095)	(0.150)	(0.157)	(0.105)	(0.206)	(0.224)
D_IB × Q2/2020				0.143	− 0.297**	− 0.205	0.240**	− 0.227	− 0.021
				(0.101)	(0.133)	(0.155)	(0.113)	(0.206)	(0.214)
D_IB × Q3/2020				0.107	0.015	0.143	0.212	− 0.122	0.112
				(0.155)	(0.143)	(0.202)	(0.184)	(0.134)	(0.195)
D_IB × Q4/2020				0.026	0.240	0.299	0.095	0.117	0.025
				(0.078)	(0.170)	(0.200)	(0.094)	(0.190)	(0.223)
D_IB × Q1/2021				− 0.144	0.408*	0.390*	− 0.085	0.261	0.167
				(0.105)	(0.221)	(0.214)	(0.122)	(0.229)	(0.240)
D_IB × Q2/2021				− 0.206**	− 0.189	− 0.102	− 0.112	0.080	0.114
				(0.103)	(0.257)	(0.269)	(0.104)	(0.212)	(0.238)
D_IB × Q3/2021				− 0.003	− 0.089	− 0.006	0.105	− 0.089	0.101
				(0.157)	(0.195)	(0.148)	(0.183)	(0.209)	(0.146)
VaR (t− 1)	0.234	0.250	5.355***	0.205	0.162	5.397***	2.488	− 1.185	4.324
	(1.247)	(1.507)	(1.867)	(1.245)	(1.493)	(1.867)	(2.401)	(5.312)	(3.433)
log(Z (t− 1))	0.272***	1.609***	3.421***	0.267***	1.609***	3.428***	0.102	1.545**	3.375***
	(0.085)	(0.363)	(0.456)	(0.085)	(0.360)	(0.455)	(0.146)	(0.650)	(0.834)
EQ (t− 1)	0.073	− 1.008**	− 1.802**	0.073	− 1.003**	− 1.798**	0.788	− 3.313	− 7.154**
	(0.061)	(0.450)	(0.698)	(0.061)	(0.446)	(0.696)	(0.778)	(2.688)	(3.266)
Lt debt (t− 1)	− 0.132	0.606**	0.867**	− 0.143	0.596**	0.858**	− 0.344	1.526**	1.138
	(0.255)	(0.260)	(0.431)	(0.260)	(0.262)	(0.433)	(0.409)	(0.681)	(0.892)
log(TA (t− 1))	0.216***	0.380***	0.723***	0.214***	0.385***	0.721***	0.165*	− 0.005	0.151
	(0.059)	(0.128)	(0.160)	(0.060)	(0.129)	(0.161)	(0.092)	(0.186)	(0.160)
ROA (t− 1)	− 0.321***	0.700**	− 0.068	− 0.304**	0.696**	− 0.059	− 1.258**	− 0.913	1.135
	(0.120)	(0.347)	(0.795)	(0.119)	(0.347)	(0.798)	(0.632)	(1.886)	(1.414)
Bank FE	Yes	Yes	Yes	Yes	Yes	Yes			
Time FE	Yes	Yes	Yes	Yes	Yes	Yes			
# of obs	6887	6865	6887	6887	6865	6887	2389	2388	2389
# of banks	258	258	258	258	258	258	86	86	86
adj R^2^	0.087	0.056	0.123	0.090	0.060	0.122	0.088	0.058	0.126

Panel b: Economic support initiatives						
	(1)	(2)	(3)	(4)	(5)	(6)
	CH_II	CH_NII	CH_NI	CH_II	CH_NII	CH_NI
InSu	0.040*	− 0.041	0.012			
	(0.023)	(0.047)	(0.054)			
D_IB × InSu	0.068**	0.047	0.097*			
	(0.027)	(0.035)	(0.053)			
DeRe				− 0.128***	0.044	0.034
				(0.041)	(0.072)	(0.103)
D_IB × DeRe				0.055***	0.025	0.065
				(0.020)	(0.039)	(0.044)
VaR (t− 1)	2.271	− 0.833	4.882	2.100	− 0.934	4.623
	(2.652)	(5.392)	(3.578)	(2.619)	(5.375)	(3.559)
log(Z(t− 1))	0.132	1.545**	3.394***	0.108	1.541**	3.367***
	(0.144)	(0.649)	(0.837)	(0.150)	(0.650)	(0.835)
EQ (t− 1)	0.837	− 3.412	− 7.147**	0.892	− 3.430	− 7.177**
	(0.799)	(2.744)	(3.330)	(0.818)	(2.768)	(3.344)
Lt debt (t− 1)	− 0.243	1.532**	1.188	− 0.334	1.520**	1.192
	(0.423)	(0.635)	(0.856)	(0.442)	(0.670)	(0.872)
log(TA(t− 1))	0.181*	− 0.016	0.161	0.187*	− 0.019	0.142
	(0.096)	(0.189)	(0.166)	(0.103)	(0.178)	(0.153)
ROA (t− 1)	− 1.312**	− 0.897	1.136	− 1.404**	− 0.823	1.218
	(0.624)	(1.890)	(1.399)	(0.618)	(1.958)	(1.477)
Q1/2020	− 0.202***	0.130	0.273**	− 0.144***	0.102	0.265***
	(0.055)	(0.100)	(0.107)	(0.051)	(0.085)	(0.097)
Q2/2020	− 0.428***	0.102	0.123	− 0.215**	0.006	0.096
	(0.064)	(0.099)	(0.106)	(0.082)	(0.090)	(0.124)
Q3/2020	− 0.560***	0.016	0.173	− 0.334***	− 0.088	0.133
	(0.096)	(0.093)	(0.126)	(0.109)	(0.130)	(0.175)
Q4/2020	− 0.400***	0.034	− 0.082	− 0.175**	− 0.069	− 0.123
	(0.048)	(0.123)	(0.140)	(0.087)	(0.159)	(0.201)
Q1/2021	− 0.361***	0.051	0.146	− 0.123*	− 0.057	0.101
	(0.065)	(0.141)	(0.146)	(0.073)	(0.198)	(0.262)
Q2/2021	− 0.303***	0.037	0.143	− 0.080	− 0.064	0.107
	(0.057)	(0.148)	(0.156)	(0.066)	(0.136)	(0.179)
Q3/2021	− 0.307***	− 0.119	0.127	− 0.114	− 0.207	0.103
	(0.093)	(0.132)	(0.109)	(0.095)	(0.145)	(0.149)
Bank FE	Yes	Yes	Yes	Yes	Yes	Yes
Time FE	Yes	Yes	Yes	Yes	Yes	Yes
# of obs	2389	2388	2389	2389	2388	2389
# of banks	86	86	86	86	86	86
adj R^2^	0.086	0.055	0.132	0.088	0.055	0.132

This table presents results from TWFE estimations on the change in finance/interest income (columns (1), (4) and (7)), non-finance/non-interest income (columns (2), (5) and (8)), and net income after taxes and provisions (columns (3), (6) and (9)). *D_IB* equals one for Islamic banks. *InSu* denotes the income support indicator, and *DeRe* is the debt relief indicator. The sample spans observations from Q3 2014 to Q3 2021. Q4 2019 is used as the base quarter. Columns (1)–(6) of Panel a are based on all Islamic and conventional banks, while in columns (7)–(9) of Panel a and in Panel b, only Islamic and conventional banks are considered that are propensity-score matched. For further variable definitions, see Appendix. The dependent variables are truncated at the 0.5 and 99.5 percentiles. Standard errors are in parenthesis and are clustered at the bank level. *, **, and *** denote statistical significance at the 10%, 5%, and 1% levels, respectively

The changes in income are influenced by several of our control variables. A higher z-score (*Z*), which means lower risk, is positively associated with the finance/interest income, non-finance/non-interest income, and net income. The equity ratio (*EQ*) correlates negatively and significantly with non-finance/non-interest income and net income. Size, measured by the logarithm of total assets (log(*TA*)), loads positively for all income positions considered. Higher return on assets in the previous quarter (*ROA*) comes with lower changes in finance/interest income but with higher changes in non-finance/non-interest income.

To investigate whether the change in income differs between Islamic and conventional banks, we interact the time-fixed effects with *D_IB*. Panel a of Table 4 reports the results in columns (4) to (6) for the full sample and in columns (7) to (9) for the propensity-score matched sample. In a few COVID-19 quarters, Islamic banks’ income changes differ from the ones of conventional banks. However, once we use our matched sample, these differences are no longer observable. For the matched sample, we can only report that in the second COVID-quarter (Q2/2020) changes in the finance income of Islamic banks are higher than the respective changes in the interest income of conventional banks (column (7)). Neither for non-finance income/non-interest income (column (8)) nor for net income (column (9)) we see any differences.^10^ Thus, overall Islamic banks perform similar to propensity-score matched conventional peers during these quarters.

To see whether Islamic banks’ income positions change as much as conventional banks’ income positions to the introduction of economic support schemes, we apply the model depicted in Eq. (2) to the matched sample.^11^ Results for income support are presented in columns (1) to (3) of Panel b, Table 4. Income support has a positive effect on finance/interest income as the positive coefficient on *InSu* in column (1) indicates. Islamic banks benefit from these income subsidies even more than conventional banks, because the coefficient on the interaction term (*D_IB* × *InSu*) is positive and significant for finance/interest income (column (1)). While income support does not affect the changes in conventional banks’ net income, it does increase the changes in Islamic banks’ net income as indicated by the significant coefficient on the interaction term (*D_IB* × *InSu*) in column (3). Thus, Islamic banks benefit more from income support schemes than their conventional counterparties.

Results for debt relief are presented in columns (4) to (6) of Panel b, Table 4. From the coefficients we are interested in, we find only two to be significant. We observe that debt relief programs (*DeRe*) come with lower finance/interest income (column (4)) and this effect is less negative for Islamic banks as the positive coefficient on the interaction term (*D_IB* × *DeRe*) indicates. This negative effect is likely driven by the fact that many debt relief programs were enacted at the very beginning of the pandemic, where uncertainty was very high. Thus, this negative effect might stem from confounding events, such as workplace closure recommendations. The correlation between workplace closure recommendations and debt relief is substantially higher than the correlation between workplace closure recommendations and income support (see, Table 3). Unfortunately, within the TWFE specifications, we cannot control for confounding events because of the high correlations between the various indicators. However, we control for confounding events in our analysis of stock price responses.

**Fig. 3:**
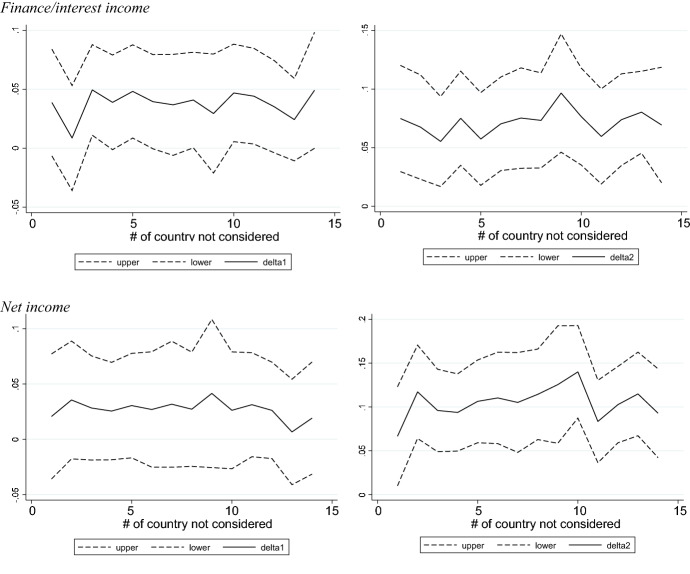
This figure presents the coefficients and their respective 90% confidence intervals on income support (*δ*_*1*_ in Eq. (2)) and on the interaction term between income support and *D_IB* (*δ*_*2*_ in Eq. (2)) when each country is eliminated once from the estimation. The dependent variable is the change in finance/interest income in the upper graphs and the change in net income in the lower graphs. The matched sample is used

We carry out two robustness tests. First, we use a 1:4 instead of a 1:1 matching approach and find the results of economic support schemes still hold. These results are available upon request. Second, we check whether banks that reside in particular countries are more relevant for the positive effects of income support schemes. For this test, we run the specifications in columns (1) and (3) of Panel b, Table 4, and exclude each country once. Since the test is performed on the matched sample, we consider only 14 countries. Figure 3 depicts the coefficients and their respective 90% confidence intervals on income support [${\delta }_{1}$ in Eq. (2)] and on the interaction term between income support and *D_IB *[${\delta }_{2}$ in Eq. (2)] when the dependent variable is either the change in finance/interest income or the change in net income. The coefficients on income support are always positive and significant for the change in finance/interest income, while they are always insignificant for net income confirming our previous results. Moreover, the coefficients on the interaction terms between income support and *D_IB* are always positive and significant for the change in finance/interest income as well as net income. This once again confirms our previous results. Thus, we conclude that the effect of income support is not driven by a single country.

### The role of PLS contracts

Islamic banks’ PLS financing contracts may be a moderating factor on how economic support schemes affect bank income as these contracts give rise to the moral hazard behavior of the borrower. For all Islamic banks, we first search Refinitiv for subcategories of Islamic income. Because the database offers the required information for a few banks only, we then hand-collect information on their income structure from their 2017 and 2018 annual reports to develop an indicator on how often these banks employ PLS arrangements. For 34 Islamic banks, we identify details of their income structure; we present average values of selected positions relative to total income in Table 5. *Musharaka* income accounts for 7.05%, while *Mudaraba* income accounts for only 3.18%. Much more relevant in terms of contribution to income are alternative loan contracts: *Murabaha* income accounts for more than 35.35%, *Ijara* income equals 18.96%, while *Istisna* income is neglectable. Taken together, income from PLS arrangements accounts, on average, for about 10.23% of the total income only. This is in line with the literature that indicates that PLS income is less important than non-PLS income. For instance, non-PLS financing to total financing made by Islamic banks in Gulf Cooperation Council countries accounted for 85.27% and generated 70% of Islamic banks’ income during the period 2002–2008 (Matoussi and Grassa 2010). Even if not all Islamic banks provide detailed income structures, many provide insight on whether they employ PLS arrangements in general. Therefore, we generate a dummy variable, *D_PLS*, which equals one if the Islamic bank employs PLS arrangements and zero otherwise. Because this dummy is available for as many as 43 Islamic banks, we use it in our next analysis to test whether Islamic banks using PLS arrangements on the asset-side of their balance sheet respond more or less to the COVID-19 economic support initiatives.

**Table 5:** Islamic income

Income position	# obs	Mean	SD
*PLS contracts*			
Musharaka income	34	7.047%	18.006%
Mudaraba income	34	3.179%	9.890%
*Non-PLS contracts*			
Murabaha income	34	35.348%	33.741%
Ijara income	34	18.964%	25.949%
Istisna income	34	0.228%	0.570%
PLS income	34	10.225%	20.141%
D_PLS	43	48.837%	

This table states the mean and standard deviation (SD) of Islamic income from financial products on the asset-side of their balance sheet before the pandemic started. The information is the average of the years 2017 and 2018. Data come from Refinitiv and are manually collected from Islamic banks’ annual reports

In Table 6, Panel a we restrict the sample to Islamic banks, apply the model depicted in Eq. (2) and replace *D_IB* with *D_PLS*. Finance income (column (1)) and non-finance income (column (2)) do not differ with the use of PLS arrangements. However, for net income (column (3)), we find a negative and significant coefficient on the interaction term between income support and *D_PLS* indicating a worse performance of Islamic banks employing PLS arrangements. The results indicate that Islamic banks employing PLS arrangements do not benefit as much from higher income support than Islamic banks not employing these arrangements. We find a similar pattern for the debt relief schemes. In column (6), the coefficient on the interaction term between debt relief and *D_PLS* indicates that Islamic banks employing PLS arrangements do worse than Islamic banks not employing PLS contracts with this support.

**Table 6:** Asset-side PLS contracts and income

Panel a: Islamic banks only						
	(1)	(2)	(3)	(4)	(5)	(6)
	CH_II	CH_NII	CH_NI	CH_II	CH_NII	CH_NI
InSu	0.096**	0.031	0.142**			
	(0.043)	(0.053)	(0.065)			
D_PLS × InSu	− 0.040	− 0.077	− 0.122*			
	(0.050)	(0.047)	(0.065)			
DeRe				− 0.090	0.047	0.097
				(0.091)	(0.112)	(0.106)
D_PLS × DeRe				− 0.017	− 0.085	− 0.116**
				(0.042)	(0.065)	(0.050)
VaR (t− 1)	3.646	− 2.983	1.817	3.333	− 2.891	1.534
	(3.670)	(6.859)	(4.513)	(3.689)	(6.798)	(4.616)
log(Z(t− 1))	0.045	1.148**	2.489***	0.007	1.171**	2.474***
	(0.232)	(0.483)	(0.475)	(0.250)	(0.505)	(0.482)
EQ (t− 1)	− 0.201	− 3.471	− 5.100***	− 0.081	− 3.466	− 5.062***
	(0.844)	(2.314)	(1.523)	(0.926)	(2.317)	(1.476)
Lt debt (t− 1)	− 2.140**	0.513	− 0.537	− 2.500***	0.368	− 0.877
	(0.837)	(0.723)	(0.710)	(0.857)	(0.712)	(0.851)
log(TA(t− 1))	0.238***	− 0.061	0.294***	0.248***	− 0.048	0.297***
	(0.073)	(0.164)	(0.096)	(0.073)	(0.160)	(0.098)
ROA (t− 1)	− 1.871***	− 2.171***	− 0.160	− 1.940***	− 2.116**	− 0.049
	(0.537)	(0.801)	(0.338)	(0.572)	(0.882)	(0.390)
Q1/2020	− 0.150	0.042	0.194*	− 0.100	0.039	0.199*
	(0.103)	(0.114)	(0.108)	(0.103)	(0.097)	(0.113)
Q2/2020	− 0.302***	− 0.037	0.032	− 0.089	− 0.051	0.066
	(0.101)	(0.114)	(0.109)	(0.161)	(0.111)	(0.159)
Q3/2020	− 0.439**	− 0.073	0.146	− 0.216	− 0.083	0.176
	(0.166)	(0.126)	(0.174)	(0.209)	(0.153)	(0.219)
Q4/2020	− 0.355***	0.059	− 0.170	− 0.133	0.048	− 0.143
	(0.075)	(0.152)	(0.142)	(0.160)	(0.157)	(0.165)
Q1/2021	− 0.402***	0.088	0.047	− 0.171	0.080	0.077
	(0.100)	(0.165)	(0.120)	(0.130)	(0.186)	(0.223)
Q2/2021	− 0.343***	0.055	0.085	− 0.120	0.044	0.116
	(0.081)	(0.182)	(0.140)	(0.126)	(0.133)	(0.145)
Q3/2021	− 0.226	− 0.161	0.109	− 0.035	− 0.177	0.135
	(0.177)	(0.204)	(0.121)	(0.203)	(0.199)	(0.156)
Bank FE	Yes	Yes	Yes	Yes	Yes	Yes
Time FE	Yes	Yes	Yes	Yes	Yes	Yes
# of obs	1189	1189	1189	1189	1189	1189
# of banks	43	43	43	43	43	43
adj R^2^	0.042	0.091	0.139	0.042	0.092	0.138

Panel b: Islamic banks with matched conventional banks						
	(1)	(2)	(3)	(4)	(5)	(6)
	CH_II	CH_NII	CH_NI	CH_II	CH_NII	CH_NI
InSu	0.041*	− 0.039	0.016			
	(0.023)	(0.047)	(0.054)			
D_IB×InSu if D_PLS=1	0.049*	0.001	0.009			
	(0.026)	(0.034)	(0.061)			
D_IB×InSu if D_PLS=0	0.086**	0.095*	0.188**			
	(0.041)	(0.053)	(0.081)			
DeRe				− 0.125***	0.054	0.050
				(0.042)	(0.075)	(0.105)
D_IB×DeRe if D_PLS=1				0.044*	− 0.032	− 0.020
				(0.024)	(0.027)	(0.049)
D_IB×DeRe if D_PLS=0				0.070**	0.096	0.169**
				(0.031)	(0.078)	(0.069)
VaR (t− 1)	2.305	− 0.748	5.043	2.131	− 0.779	4.851
	(2.660)	(5.388)	(3.534)	(2.602)	(5.468)	(3.539)
log(Z(t− 1))	0.141	1.566**	3.433***	0.116	1.581**	3.427***
	(0.148)	(0.655)	(0.840)	(0.155)	(0.662)	(0.843)
EQ (t− 1)	0.825	− 3.444	− 7.208**	0.885	− 3.464	− 7.228**
	(0.808)	(2.755)	(3.332)	(0.822)	(2.761)	(3.324)
Lt debt (t− 1)	− 0.238	1.545**	1.211	− 0.337	1.503**	1.168
	(0.427)	(0.635)	(0.857)	(0.444)	(0.655)	(0.864)
log(TA(t− 1))	0.182*	− 0.014	0.165	0.188*	− 0.012	0.153
	(0.097)	(0.188)	(0.166)	(0.103)	(0.175)	(0.150)
ROA (t− 1)	− 1.316**	− 0.908	1.115	− 1.395**	− 0.778	1.284
	(0.630)	(1.899)	(1.410)	(0.622)	(1.977)	(1.495)
Q1/2020	− 0.200***	0.133	0.281**	− 0.144***	0.103	0.266***
	(0.055)	(0.101)	(0.108)	(0.051)	(0.086)	(0.098)
Q2/2020	− 0.430***	0.096	0.111	− 0.220***	− 0.017	0.062
	(0.063)	(0.098)	(0.105)	(0.082)	(0.094)	(0.124)
Q3/2020	− 0.561***	0.013	0.168	− 0.338***	− 0.105	0.108
	(0.096)	(0.092)	(0.125)	(0.108)	(0.135)	(0.176)
Q4/2020	− 0.402***	0.031	− 0.088	− 0.178**	− 0.088	− 0.150
	(0.048)	(0.123)	(0.141)	(0.088)	(0.163)	(0.204)
Q1/2021	− 0.362***	0.048	0.139	− 0.127*	− 0.076	0.074
	(0.064)	(0.140)	(0.145)	(0.072)	(0.203)	(0.265)
Q2/2021	− 0.304***	0.034	0.136	− 0.084	− 0.083	0.080
	(0.057)	(0.148)	(0.157)	(0.066)	(0.136)	(0.179)
Q3/2021	− 0.309***	− 0.123	0.118	− 0.118	− 0.227	0.073
	(0.092)	(0.132)	(0.109)	(0.094)	(0.149)	(0.151)
Bank FE	Yes	Yes	Yes	Yes	Yes	Yes
Time FE	Yes	Yes	Yes	Yes	Yes	Yes
# of obs	2389	2388	2389	2389	2388	2389
# of banks	86	86	86	86	86	86
adj R^2^	0.086	0.055	0.134	0.087	0.057	0.134

This table presents results from TWFE estimations. In Panel a, only Islamic banks are considered, while in Panel b, the matched sample of Islamic and conventional banks is used. *D_PLS* denotes a dummy variable equal to one if an Islamic bank employs PLS arrangements in its finance products before the pandemic started. In columns (1) and (4) [(2) and (5)] the dependent variable is the change in finance income/interest income [non-finance income /non-interest income], and in columns (3) and (6), it is the change in the net income after taxes and provisions. The sample spans Q3 2014 to Q3 2021. Q4 2019 is used as the base quarter. For further variable definitions, see Appendix. The dependent variables are truncated at the 0.5 and 99.5 percentiles. Standard errors are in parenthesis and are clustered at the bank level. *, **, and *** denote statistical significance at the 10%, 5%, and 1% levels, respectively

In Table 6, Panel b we use the matched sample and extent the model depicted in Eq. (2) by including two interaction terms, one to measure the effect of economic support initiatives for PLS employing Islamic banks and the other is for Islamic banks not using PLS contracts. Income support fosters non-PLS employing Islamic banks’ non-finance as well as net income while debt relief stimulates their net income only. Overall, our evidence suggests that the net income of Islamic banks using PLS arrangements is not as much fostered by these economic support programs as the one of non-PLS employing banks. While one may argue that this could signal agency problems stirred by the support programs, we do not observe this pattern in the finance income. Thus, we have no evidence in line with an agency problem fostered by economic support programs to be at work for PLS employing Islamic banks.

## Stock price response

In this section, we present our results on how banks’ stock prices change when COVID-19 measures take effect. We first discuss our methodology and afterward present the results from event-study tests and multivariate analysis.

### Methodology

We use traditional event-study methodology (e.g., Campbell et al. 2010; MacKinlay 1997; Brown and Warner 1985) to examine the effect that the COVID-19 pandemic has on the stock price of Islamic and conventional banks. We consider the dates of the first workplace closure, the first income support action, and the first debt relief action as our event dates. Workplace closures are usually announced several days before the closures take effect. For this event, we are therefore interested in the response of banks’ stock prices before the effective date. Income support and debt relief measures, however, are announced and take effect immediately; for these types of events, we are interested in the response of stock prices around the effective date.

We follow prior literature and utilize the market model (Campbell et al. 2010) to determine the abnormal return of a bank on the measure’s effective date. Because some of the stocks in our sample are thinly traded, we also consider lagged effects of market return movements in our estimation model. The model looks like this:

3 $${R}_{id}= {\alpha }_{i}+ {\beta }_{i}^{0}{R}_{md}+{\beta }_{i}^{1}{R}_{md-1}+{\beta }_{i}^{2}{R}_{md-2}+{\varepsilon }_{id}$$

where ${R}_{id}$ is the return of bank stock *i* on day *d*, ${R}_{md}$ is the return of the country-specific market index on day *d*, ${\varepsilon }_{id}$ is the error term, and ${\alpha }_{i}$ and ${\beta }_{i}$ are the unknown parameters of the market model. These parameters are estimated using an estimation window of 252 days before the event window which ends 21 days before the event.

With the information from this market model, we calculate abnormal returns as the difference between the actual and the normal return:

4 $$A{R}_{i\tau }={R}_{i\tau }-{\widehat{\alpha }}_{i}-{\widehat{\beta }}_{i}^{0}{R}_{m\tau }-{\widehat{\beta }}_{i}^{1}{R}_{m\tau -1}-{\widehat{\beta }}_{i}^{2}{R}_{m\tau -2}$$

where ${AR}_{i\tau }$ is the abnormal return of bank *i* on day *τ*, and ${\widehat{\alpha }}_{i}$ and ${\widehat{\beta }}_{i}$ are the estimated parameters of the market model for bank *i*. *τ* denotes the event time, it is equal to zero on the event day and + 1/-1 on the day after/before the event takes place.

Cumulative abnormal returns (*CARs*) cumulate the abnormal returns from day ${\tau }_{1}$ up to day ${\tau }_{2}:$

5 $${CAR}_{i}\left({\tau }_{1},{\tau }_{2}\right)=\sum_{{\tau }_{1}}^{{\tau }_{2}}{AR}_{i\tau }.$$

### Event-study test and multivariate analysis

**Fig. 4:**
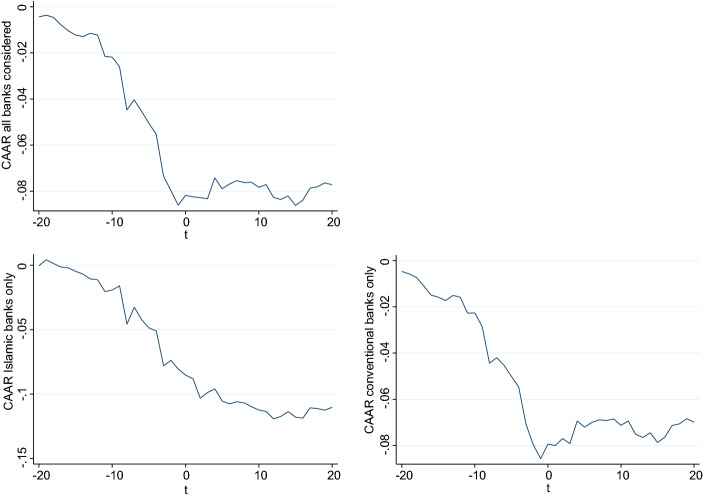
This figure presents cumulative average abnormal returns (*CAARs*) of banks in dual-banking countries around workplace closures in spring 2020. Abnormal returns are cumulated from 20 days before the event to 20 days after. We determine normal returns by using a domestic-factor model with thin trading adjustment and estimate parameters to calculate normal returns from the 252-trading-day period ending 21 trading days before the workplace closure

In Fig. 4, we plot the cumulative average abnormal returns (*CAARs*) of all banks, Islamic banks, and conventional banks starting 20 days before the effective date of the countries’ first workplace closures up to 20 days after this date. As expected, stock prices start to decline before the effective date of the workplace closure. While the *CAAR* of Islamic banks continuously declines between 20 days before the workplace closure and the day it takes effect, the *CAAR* of conventional banks declines moderately in the first ten days of the event window and sharply afterward.

To determine the statistical significance of the *CAARs,* we utilize parametric and nonparametric tests. As for the parametric test, we employ the test proposed by Kolari and Pynnönen (2010), because it accounts for cross-sectional dependencies, which is relevant in our setting as a country’s workplace closure affects all banks located in this country at the same point in time. We further apply the nonparametric generalized sign test (Cowan 1992), which is more powerful than the two parametric tests that Campbell et al. (2010) consider in a multicountry event-study context such as ours.

In Table 7, Panel a, we present *CAAR*s from several event windows calculated either with a simple market model or a market model that adjusts for thin trading according to Eq. (4). Our preferred event window starts ten days before the workplace closure and ends on the day of the workplace closure. This *CAAR* is − 6.06% when we tackle thin trading in the normal return prediction and − 7.22% when it is not considered. This difference indicates that predicting normal returns without controlling for thinly traded stocks may overestimate the effect that workplace closures have on bank stocks. Therefore, we apply the model with the adjustment for thin trading in the rest of our analysis. Overall, our stock price responses from banks in dual-banking countries are broadly in line with the findings from other studies. Demirgüç-Kunt et al. (2021) show that banks headquartered in industrial and developing countries significantly lost more market value than nonfinancial firms in the first quarter of 2020.

**Table 7:** Abnormal bank returns around the effective dates of COVID-19 measures

Panel a: Workplace closures in dual-banking countries						
[τ1,τ2]	Market model with thin trading			Market model		
	# obs 210			210		
	CAAR	KP	C	CAAR	KP	C
[− 10,0]	− 6.059%	***	***	− 7.221%	***	***
[− 5,0]	− 4.665%	***	***	− 5.696%	***	***
[− 20,− 10]	− 2.152%	**	**	− 2.564%	***	***
[− 20,0]	− 8.690%	***	***	− 10.203%	***	***
[− 1,1]	− 1.020%	*		− 1.481%	**	*

Panel b: Workplace closures							
[τ1,τ2]	Islamic banks			Conventional banks			Equality test
	# obs 35			175			
	CAAR	KP	C	CAAR	KP	C	
[− 10,0]	− 5.746%	***	**	− 6.017%	***	***	0.883
[− 5,0]	− 3.481%	**	**	− 4.892%	***	***	0.376
[− 20,− 10]	− 1.473%			− 2.219%	**	**	0.466
[− 20,0]	− 7.684%	***	***	− 8.733%	***	***	0.626
[− 1,1]	− 1.359%			− 0.958%	*		0.743

Panel c: Income support							
[τ1,τ2]	Islamic banks			Conventional banks			Equality test
	# obs 26			149			
	CAAR	KP	C	CAAR	KP	C	
[− 10,0]	2.632%		**	1.891%			0.474
[− 5,0]	0.306%			0.478%			0.703
[− 1,1]	0.857%	**	*	0.011%			0.017

Panel d: Debt relief							
[τ1,τ2]	Islamic banks			Conventional banks			Equality test
	# obs 35			175			
	CAAR	KP	C	CAAR	KP	C	
[− 10,0]	− 0.216%			− 2.156%	**	**	0.174
[− 5,0]	− 0.314%			− 1.194%	*		0.481
[− 1,1]	− 0.312%			0.691%			0.404

This table shows the cumulative average abnormal return *CAAR*[*τ*_1_,*τ*_2_] of banks located in dual-banking countries when workplaces are closed for the first time due to the COVID-19 pandemic in Panels a and b, when the first income support takes effect in Panel c, and when the first debt relief is enacted in Panel d. Average abnormal returns are cumulated over *τ*_*1*_ to *τ*_*2*_, where *τ* = 0 is the effective date of the respective COVID-19 measure. # obs refers to the number of observations we consider in the calculation of *CAAR.* We determine normal returns by employing a domestic-factor model with or without adjustment for thin trading and estimate parameters to predict normal returns from the 252-trading-day period ending 21 trading days before the event. In Panel a, we consider all banks and in Panels b to d we split banks into Islamic and conventional banks and employ the domestic-factor model with adjustment for thin trading. *KP* reports statistical significance levels based on the parametric test statistic proposed by Kolari and Pynnönen (2010), while *C* comes from the nonparametric generalized sign test according to Cowan (1992). In Panels b-d, equality tests report significance from rank tests between Islamic and conventional banks. ***, ** and * denote statistical significance at the 1%, 5% and 10% levels, respectively

A comparison of the various event windows suggests that the main drop in stock prices occurs within the two weeks before the closure. The *CAAR* from 20 to 10 days before the event accounts for − 2.15%, while the *CAAR* from five days before to the day of the closure is as high as − 4.67%, which means the latter effect is double as high as the first one but contains only six instead of 11 days. The *CAAR* that contains the day before and after the workplace closure is less negative and only weakly significant. Overall, these results indicate that banks lose much more value with the workplace closure than the banks’ respective domestic stock markets.

In Panel b, we distinguish between Islamic and conventional banks to see whether they similarly respond to the workplace closure. For Islamic banks, the *CAAR*[− 10,0] is − 5.75%, while the one of the conventional banks is − 6.02%. Also, for the window from 20 days before to the day of the workplace closure, the abnormal return is less negative for Islamic banks than for conventional banks. However, cross-sectional tests indicate that the *CAAR*[− 10,0] for Islamic banks does not differ significantly from the one of conventional banks. Moreover, cross-sectional tests for all other event windows considered indicate that *CAARs* of Islamic banks do not differ significantly from the ones of conventional banks. Overall, this evidence suggests that the stock prices of Islamic and conventional banks are similarly hit by the COVID-19 workplace closure.

Our abnormal returns may differ from the ones reported in the literature. For instance, Mirazae et al. (2021) find that the stock returns of Islamic banks are about 10–13% higher than those of conventional banks during the initial phase of the COVID-19 crisis. This difference in stock price responses has several potential sources: (i) we employ event-study methodology and focus on the 4 weeks before the workplace closure, which we believe to be the severest event in the initial phase of the pandemic, while other researchers focus on the first quarter (e.g., Demirgüç-Kunt et al. 2021), and (ii) we exclude insufficiently traded stocks and consider adjustments due to thin trading in our model predicting normal returns, while other researchers do not (Mirazae et al. 2021).

We also report test results on how banks’ stock prices change when the first income support (Panel c), and the first debt relief (Panel d) is announced. With respect to the first income support, the *CAAR*[− 1,1] is 0.86% for Islamic banks, which is significantly different from zero as indicated by the parametric and nonparametric tests, while it is 0.01% for conventional banks. A cross-sectional test indicates that the stock price changes to income support announcements of both bank types differ significantly from each other. Thus, Islamic banks’ stock prices are more positively affected than conventional banks when income support is introduced. Concerning the first debt relief, the *CAAR*[− 1,1] is − 0.31% for Islamic banks and 0.69% for conventional banks. However, both numbers lack statistical significance, and a cross-sectional test indicates that they do not differ from each other. Using a worldwide sample of banks, Demirgüç-Kunt et al. (2021) document significantly positive abnormal returns for the announcement of borrower assistance programs. This finding is not necessarily in contrast to our result, because Demirgüç-Kunt et al. (2021) further show that large banks benefit much more than small banks, and most banks in dual-banking countries belong to the group of small banks.

We next use a multivariate analysis that allows us to control for much more factors that may moderate the stock price response. Our model is as follows:

6 $${CAR}_{i}[{\tau }_{1},{\tau }_{2}]=\alpha +\gamma \times {D\_IB}_{i}+{\beta }_{1}\times {D\_INVESTMENT}_{i}+{\beta }_{2}\times {D\_LARGE}_{i}+{\beta }_{3}\times {VaR}_{it-1}+{\beta }_{4}\times {\mathrm{log}(Z}_{it-1})+{\beta }_{5}\times {EQ}_{it-1}+{\beta }_{6}\times {Lt debt}_{it-1}+{\beta }_{7}\times {\mathrm{log}(TA}_{it-1})+{\beta }_{8}\times {ROA}_{it-1}+{\lambda }_{t}+{\mu }_{Country}{+\varepsilon }_{i}$$

where ${CAR}_{i}$ denotes the cumulative abnormal return of bank *i*. *D_IB* is the variable of interest and denotes a dummy variable that is set to one for Islamic banks. We consider a fixed effect for each country, ${\mu }_{Country}$, which controls for all country-invariant characteristics, such as the severity of the pandemic in a country. As in the previous section, we use heteroscedasticity-consistent standard errors clustered at the bank level.

In Panel a of Table 8, we present the results on workplace closures for the *CAR*[− 10,0] and *CAR*[− 5,0]. In all specifications of the full sample (columns (1) to (3)), the dummy variable for Islamic banks is insignificant, which is in line with our findings in Table 7. When we compare Islamic banks to conventional banks with similar characteristics (columns (4) and (5)), the dummy variable for Islamic banks is also insignificant. Several of our control variables moderate how the workplace closure affects the stock price responses. Abnormal returns of banks increase with investment banking services (D_*INVESTMENT*) and higher Z-scores (*Z*) and decrease with higher long-term debt (*LT debt*) and higher return on assets (*ROA*). In Panel b, we focus on economic support schemes by combining the announcement returns of the first income support and debt relief. The announcement of the first income support (*Event_IS*) comes with a lower *CAR*[− 1,1] than the announcement of the first debt relief. This effect is significant when we include controls (column (2)), but not when controls are not considered (column (1)). Next, we test whether Islamic banks’ *CAR*s are moderated by the type of economic support initiative. In column (3), we find that Islamic banks’ *CAR*s are significantly higher when income support (*D_IB* × *Event_IS*) is announced confirming the results of Table 7. Thus, Islamic banks gain more value than their conventional peers when income support measures are announced. These findings might be driven by confounding events, which may have severe effects on the outcome (Jong and Naumovska 2016). Therefore, we next exclude *CAR*s that occur on the same day as debt relief as well as workplace closure actions (see Table 2). Column (4), which comes from the sample where confounding events are excluded, confirms that Islamic banks’ stock prices respond more positively to the income support actions than the stock prices of their conventional peers. In column (5), we see that this result also holds when confounding events are excluded from the propensity-score matched sample.

**Table 8:** *CAR* analysis

Panel a: Workplace closure					
	(1)	(2)	(3)	(4)	(5)
	All	All	All	Matched	Matched
	CAR[− 10,0]	CAR[− 10,0]	CAR[− 5,0]	CAR[− 10,0]	CAR[− 5,0]
D_IB	0.008	0.018	0.024	− 0.014	0.003
	(0.017)	(0.020)	(0.016)	(0.034)	(0.023)
D_INVESTMENT		0.072***	0.059***	− 0.005	0.000
		(0.017)	(0.015)	(0.023)	(0.022)
D_LARGE		0.013	− 0.000		
		(0.020)	(0.021)		
VaR (t− 1)		0.136	0.553	− 1.827	− 0.101
		(1.186)	(1.060)	(3.797)	(3.166)
log(Z (t− 1))		0.024*	0.022*	− 0.055	− 0.033
		(0.013)	(0.012)	(0.043)	(0.030)
Lt debt (t− 1)		− 0.003**	− 0.002*	− 0.011**	− 0.006
		(0.001)	(0.001)	(0.005)	(0.004)
EQ (t− 1)		0.056	0.082	− 0.546	− 0.338
		(0.104)	(0.082)	(0.337)	(0.317)
log (TA (t− 1))		0.000	0.007	0.016	0.021
		(0.006)	(0.006)	(0.025)	(0.020)
ROA (t− 1)		− 0.880*	− 0.793**	0.554	0.307
		(0.515)	(0.398)	(1.523)	(1.261)
Country FE	Yes	Yes	Yes	Yes	Yes
# of obs	210	184	184	66	66
adj R^2^	0.081	0.190	0.176	0.182	0.292

Panel b: Income support and debt relief					
	(1)	(2)	(3)	(4)	(5)
	All	All	All	All	Matched
	CAR[− 1,1]	CAR[− 1,1]	CAR[− 1,1]	CAR[− 1,1]	CAR[− 1,1]
D_IB	− 0.002	− 0.002	− 0.008	− 0.009	− 0.019*
	(0.004)	(0.004)	(0.005)	(0.005)	(0.011)
Event_IS	− 0.006	− 0.007*	− 0.009***	− 0.007**	− 0.006
	(0.004)	(0.003)	(0.002)	(0.003)	(0.009)
D_IB×Event_IS			0.016**	0.017**	0.021*
			(0.007)	(0.008)	(0.012)
D_INVESTMENT		− 0.010**	− 0.010*	− 0.011*	− 0.002
		(0.005)	(0.006)	(0.005)	(0.007)
D_LARGE		0.001	0.001	0.006	
		(0.008)	(0.007)	(0.007)	
VaR (t− 1)		0.381	0.395	0.247	2.892***
		(0.493)	(0.511)	(0.490)	(0.664)
log(Z (t− 1))		− 0.008	− 0.008	− 0.010*	− 0.013*
		(0.005)	(0.006)	(0.006)	(0.007)
Lt debt (t− 1)		0.000	0.000	0.001*	− 0.002
		(0.000)	(0.000)	(0.000)	(0.001)
EQ (t− 1)		− 0.045**	− 0.045**	− 0.042**	− 0.169**
		(0.018)	(0.019)	(0.020)	(0.083)
log (TA (t− 1))		− 0.004**	− 0.004**	− 0.004**	0.001
		(0.002)	(0.002)	(0.002)	(0.004)
ROA (t− 1)		0.507***	0.508***	0.440**	1.924***
		(0.173)	(0.092)	(0.174)	(0.517)
Country FE	Yes	Yes	Yes	Yes	Yes
# of obs	385	342	342	290	79
adj R^2^	0.054	0.148	0.151	0.143	0.444

Panel c: Banking support					
	(1)	(2)	(3)	(4)	(5)
	All	All	All	All	Matched
	CAR[− 5,0]	CAR[− 5,0]	CAR[− 1,1]	CAR[− 5,0]	CAR[− 1,1]
D_IB	− 0.003	− 0.006	− 0.002	− 0.007*	− 0.001
	(0.003)	(0.004)	(0.003)	(0.004)	(0.004)
Q1	− 0.026***	− 0.026***	− 0.007	− 0.029***	− 0.025
	(0.006)	(0.007)	(0.006)	(0.009)	(0.018)
D_IB×Q1	0.004	0.005	− 0.014	0.001	0.005
	(0.013)	(0.014)	(0.012)	(0.014)	(0.018)
D_INVESTMENT		0.010**	0.004	0.011**	0.004
		(0.004)	(0.003)	(0.005)	(0.003)
D_LARGE		− 0.001	0.001	− 0.000	
		(0.006)	(0.003)	(0.006)	
VaR (t− 1)		− 0.180	− 0.075	− 0.335	0.201
		(0.438)	(0.334)	(0.523)	(0.423)
log(Z (t− 1))		0.001	− 0.003	− 0.001	0.003
		(0.004)	(0.002)	(0.004)	(0.003)
Lt debt (t− 1)		− 0.001***	− 0.000	− 0.001***	− 0.000
		(0.000)	(0.000)	(0.000)	(0.001)
EQ (t− 1)		− 0.014	0.008	− 0.013	0.059*
		(0.031)	(0.020)	(0.035)	(0.032)
log (TA (t− 1))		− 0.002	− 0.001	− 0.003	0.002
		(0.002)	(0.001)	(0.002)	(0.004)
ROA (t− 1)		− 0.180	− 0.081	− 0.175	− 0.753***
		(0.205)	(0.107)	(0.228)	(0.245)
Country FE	Yes	Yes	Yes	Yes	Yes
# of obs	1292	1192	1064	1064	319
adj R^2^	0.115	0.116	0.080	0.127	0.161

Panel d: PLS arrangements					
	(1)	(2)	(3)	(4)	(5)
	Workplace closure		Income support	Debt relief	Banking support
	CAR[− 10,0]	CAR[− 5,0]	CAR[− 1,1]	CAR[− 1,1]	CAR[− 5,0]
D_PLS	0.002	− 0.006	− 0.000	0.009	0.006
	(0.032)	(0.026)	(0.008)	(0.012)	(0.009)
Q1					− 0.030***
					(0.010)
D_PLS×Q1					− 0.011
					(0.031)
D_INVESTMENT	0.024	0.036	0.003	− 0.003	0.007
	(0.027)	(0.023)	(0.007)	(0.010)	(0.008)
VaR (t− 1)	− 0.907	2.410	− 0.537	3.549	− 0.002
	(7.241)	(3.958)	(1.245)	(2.424)	(0.916)
log(Z (t− 1))	− 0.009	0.031	0.024	0.002	0.010
	(0.039)	(0.028)	(0.020)	(0.017)	(0.015)
Lt debt (t− 1)	− 0.002	0.001	− 0.003	0.000	− 0.001
	(0.006)	(0.003)	(0.002)	(0.002)	(0.002)
EQ (t− 1)	0.086	0.197	− 0.066	0.067	− 0.089
	(0.419)	(0.237)	(0.124)	(0.124)	(0.067)
log (TA (t− 1))	− 0.026	0.000	− 0.008	− 0.001	− 0.002
	(0.028)	(0.015)	(0.009)	(0.009)	(0.006)
ROA (t− 1)	1.958	0.884	− 1.238	1.479	− 0.143
	(1.852)	(1.063)	(0.819)	(0.992)	(0.443)
Country FE	Yes	Yes	Yes	Yes	Yes
# of obs	34	34	24	34	200
adj R^2^	0.096	− 0.054	0.108	0.190	0.053

Results are generated from ordinary least square regressions. The dependent variable in Panel a is the *CAR* when the first workplace closure takes effect. In Panel b, it is the *CAR* when the first income support or the debt relief was implemented. In Panel c, it is the *CAR* when banking-relevant measures take effect. In Panel d, we consider only Islamic banks’ *CARs* for the workplace closure (columns (1) and (2)), the income support (column (3)), the debt relief (column (4)), and banking-relevant measures (columns (5) and (6)). Columns based on *all* come from the full sample, while columns based on *matched* consider only the matched sample. For variable definitions, see Appendix. Standard errors are in parenthesis and are clustered at the bank level. *, **, and *** denote statistical significance at the 10%, 5%, and 1% levels, respectively

Following Harjoto et al. (2021), one may argue that the first workplace closure takes place in the rising infection period, which is characterized by overreaction and the stock markets start to resettle after this phase. Therefore, the non-difference in the stock price response of Islamic and conventional banks when workplaces are closed might result from such an overreaction. To see whether the effects systematically vary between the rising and stabilizing infection periods, we follow other researchers (Demirgüç-Kunt et al. 2021; Feyen et al. 2020) and study the effects of COVID-19 measures which are directly related to the banking sector, such as liquidity support and prudential measures (Gispert et al. 2020). We present the results in Panel c of Table 8, where we include a Q1 dummy variable that equals one if a banking-sector measure took effect in the first quarter of 2020, and zero otherwise. About 27% of all banking-support events occurred in the first quarter of 2020. The coefficient on this indicator shows that bank stocks lost significantly more value in the first quarter than in other quarters of 2020 when the *CAR*[− 5,0] is used (see, columns (1), (2), and (4)). In addition to our previous control variables, we also include an interaction term between this Q1 dummy variable and *D_IB*, but we find no difference between Islamic and conventional banks, and therefore conclude that their values are similarly affected by the COVID-19 banking sector measures.

Finally, we investigate whether the use of PLS contracts moderates Islamic banks’ response to COVID-19 policy measures. In Panel d of Table 8, we consider only Islamic banks and test whether Islamic banks’ stock price response depends on whether or not they employ PLS arrangements in their finance products in the pre-pandemic years. Irrespective of the event type, we find that the stock price response of banks that employ PLS arrangements do not differ from Islamic banks that do not employ PLS contracts.

## Concluding remarks

This paper examines the effects of COVID-19 policy measures on banks’ accounting- and market-based performance in dual-banking countries over the period from 2014 to 2021. We investigate whether conventional banks’ performance experiences more or less negative changes than that of Islamic banks during the pandemic. By employing two-way fixed-effect regressions, we find that the changes in Islamic banks’ finance income, as well as net income after taxes and provisions, are similar to the changes of conventional banks’ interest income and net income in the course of the pandemic. Furthermore, we see that these two banking types’ stock prices respond similarly to the first workplace closure in this pandemic which was enacted in March 2020 in most countries. These results are confirmed when we employ a propensity-score matching approach to find a conventional bank with similar characteristics for each Islamic bank. With text-based measures from the banks’ business descriptions, we find that Islamic banks are less likely to provide investment banking services and services for large customers. Controlling these services is relevant as these business segments are expected to be hurt less from the pandemic. Overall, our findings indicate that the performance of Islamic and conventional banks is similarly affected during the pandemic.

Our analyses on economic support schemes show that income support positively affects the changes in finance/interest income, but it does not affect the changes in non-finance/non-interest as well as net income. Islamic banks’ finance income and net income increase more with income support than the changes in conventional banks’ interest income and net income. Stock prices of Islamic banks respond more positively to the announcement of government income support than the ones of conventional banks, while their responses do not differ when debt relief programs are announced. We conclude that Islamic banks’ accounting- and market-based performance benefits more from income support than the one of propensity-score-matched conventional banks. This performance increase can be explained by Islamic banks’ focus on private clients, whose debt repayment likelihood receives a strong stimulus from the income support programs.

The effects of economic support programs may be moderated by Islamic banks’ use of PLS finance contracts, which are known to be a source of agency problems. Therefore, we test whether Islamic banks using PLS arrangements in the pre-pandemic years have lower income changes than Islamic banks not employing these schemes when economic support is offered. Hand-collected information indicates that about 50% of the Islamic banks in our sample employ PLS schemes in their finance products. Our results show that the change in net income when COVID-19 support measures are in place is significantly higher for Islamic banks not employing PLS financing products than that of Islamic banks with PLS schemes. However, we do not find support to the higher agency problems in PLS finance contracts as we document that COVID-19 economic support programs similarly foster finance income of PLS and non-PLS employing Islamic banks.

As with any empirical study, our study is subject to limitations. First, we consider bank income only up to the third quarter of 2021 while it is clear that the pandemic is not yet over. Nevertheless, we believe that our analysis would provide insights as a very severe drop in income and stock prices was materialized in 2020. Second, the results on the economic support measures may suffer from confounding events, as many countries install workplace closures, income support and debt relief, and other measures at the same point in time or the same quarter. While we cannot rule out confounding events in our income analysis, we rule out these events in our analysis of stock price responses. These confounding events are also the reason that we do not implement a difference-in-differences approach on the issuance of the economic support schemes because the number of treated and untreated banks, especially Islamic banks, would be much too low. Relying on two-way fixed-effects regressions in our income analysis thus means that our results should not be interpreted in a causal way.

## Appendix: Variable definitions and sources

### Dependent variables

*II*: Income from finance-based assets if the bank operates under Islamic rules, and interest income if the bank is conventionally operated. Source: Refinitiv
*NII*: Non-finance income if the bank operates under Islamic rules, and non-interest income if the bank is conventionally operated. Source: Refinitiv
*NI*: Net income after provisions and taxes. Source: Refinitiv
*CH_Y*: The quarterly change in *Y* relative to the previous quarter’s total assets
*CAR*_*i*_*[τ*_*1*_*,τ*_*2*_*]*: Cumulative abnormal return of bank *i* when a COVID-19 measure takes effect at a point in time τ = 0 which is the effective date. Abnormal returns are cumulated over τ1 to τ2. We estimate parameters for calculating risk-adjusted normal returns from the 252-trading-day period ending 21 trading days before the event. Returns are calculated from dividend- and split-adjusted share prices in local currency. From this analysis, banks are removed if more than 50% of their daily return observations in 2019/2020 are equal to zero. *Source*: Authors’ own calculation based on data from Datastream, effective dates of workplace closures, income support, and debt relief come from Hale et al. (2020) and effective dates of banking support measures come from Gispert et al. (2020)
*CAAR[τ*_*1*_*,τ*_*2*_*]*: Cumulative abnormal returns averaged over all banks when a COVID-19 measure takes effect. It is calculated as $CAAR\left({\tau }_{1},{\tau }_{2}\right)=\frac{1}{N}\sum_{i=1}^{N}{CAR}_{i}({\tau }_{1},{\tau }_{2})$

### Independent variables

*LOCK*: Quarterly average of the daily reported workplace closure indicator. If the indicator is 0, no measures are in effect. If it is 1, a closing is recommended; if the indicator equals 2 [3] a required closing or work from home is in place for some sectors or categories of workers [all-but-essential workplaces, e.g., grocery stores, doctors]. Source: Hale et al. (2020)
*InSu*: Quarterly average of the daily reported income support indicator. The indicator takes three different values. If the indicator is 0, no measures are in effect. If it is 1, the government replaces less than 50% of lost salary; if the indicator equals 2 the government replaces 50% or more of lost salary. Source: Hale et al. (2020)
*DeRe*: Quarterly average of the daily reported debt relief indicator. This indicator captures whether the government is freezing financial obligations (e.g., stopping loan repayments). The indicator takes three different values. If the indicator is 0, no measures are in effect. If it is 1, a narrow relief is in place; if the indicator equals 2 a broad debt relief is active covering several kinds of debt contracts. Source: Hale et al. (2020)
*Event_IS*: Dummy variable equals one if income support is introduced, and zero when debt relief is enacted. Source: Hale et al. (2020)
*D_IB*: Dummy variable equals one for Islamic banks, zero for conventional banks. Source: Hand-collected information
*D_PLS*: Dummy variable equals one if an Islamic bank uses PLS arrangements in financial products belonging to its asset side of the balance sheet, zero otherwise. Source: Refinitiv and hand-collected information
*D_INVESTMENT*: Dummy variable equals one if the business description of the bank in Refinitiv contains one of the following terms: investment banking, M&A, mergers, advisory service, zero otherwise. Source: Refinitiv
*D_LARGE*: Dummy variable equals one if the business description of the bank in Refinitiv contains one of the following terms: large borrower, large customer, large client, zero otherwise. Source: Refinitiv
*D_SMALL*: Dummy variable equals one if the business description of the bank in Refinitiv contains one of the following terms: small in combination with {client, customer, borrower, business, enterprise}, SME and micro lending without that large borrowers are served, zero otherwise. Source: Refinitiv
*D_PRIVATE*: Dummy variable equals one if the business description of a bank in Refinitiv contains one of the following terms: personal mortgage, personal loan, private mortgage, private loan, residential mortgage, housing finance, zero otherwise. Source: Refinitiv
*VaR*: Value-at-risk equals the absolute value of the 10th percentile of the stock returns if negative. Source: own calculation based on data from Datastream
*SD*: Standard deviation of stock returns. Source: own calculation based on data from Datastream
*Z*: *Z* is the z-score and measures the distance from insolvency. It is calculated as z = (*ROA* + *EQ*)/SD(*ROA*), where *ROA* denotes the return on assets and *EQ* is the equity ratio. The standard deviation of *ROA* is calculated from annual data over 10 years. A higher z-score indicates that the bank is more stable
*Tier1*: Tier 1 capital ratio. Source: Refinitiv
*EQ*: Equity relative to total assets. Source: Refinitiv
*Lt debt*: Total long-term debt as a percentage of total assets. Source: Refinitiv
*TA*: Total assets. Source: Refinitiv
*ROA*: Return on assets. Source: Refinitiv.

## Footnotes

1 Conventional banks are financial institutions that charge interest on loans as part of their operations, while Islamic banks strictly follow *Shariah* and Islamic teaching in their banking products and transactions by sharing risk and profits with customers instead of charging interest.

2 Throughout the text, we use the term net income to refer to net income after taxes and provisions.

3 For Islamic banks, previous research tests whether income measures, such as the net interest margin and income diversification, help explain bank performance and stability (e.g., Molyneux and Yip 2013; Pappas et al. 2017; Alandejani et al. 2017; Alqahtani and Mayes 2018).

4 Under *Mudaraba* funding contract, depositors do not impose any restrictions as to what, where, and when to invest the funds. Instead of being paid a fixed interest rate (prohibited by Islamic law), depositors receive a slice of a bank’s profits and its losses (Wigglesworth 2009).

5 We exclude the UK as conventional banks located in this country would otherwise dominate our analysis.

6 We refer the reader to the paper by Landis and Skouras (2021), who document relevant data problems in Datastream, the data source we are using.

7 Prior studies document that Islamic banks have a different structure of assets and liabilities compared to conventional peers (Vogel and Hayes 1998; Iqbal and Mirakhor 2007; Van Greuning and Iqbal 2008).

8 This part of the analysis could be carried out on publicly listed and non-listed banks in dual-banking countries. However, data availability hinders us to extend the analysis due to the lack of quarterly data for non-listed banks. Using annual data with an extended time period after the first workplace closure has the potential to pick up other effects that might be unrelated to the COVID-19 policy measures.

9 We follow Memmel and Schertler (2013) and scale interest income by total assets because interest-earning assets as in Saunders and Schumacher (2000) are not available for the banks in our sample.

10 Noteworthy is also that the non-finance/non-interest income changes are significantly positive in the first two quarters of 2020 when all banks are included in column (5) of Panel a, Table 4, while this effect vanishes once we apply the propensity-score matching. This change in quarter effects can be explained by banks’ investment banking activities fully included in column (5) and considered to a minor extent in column (8).

11 The variance inflation factors of *InSu* and *DeRe* with the time-fixed effects of the TWFE specification are 2.5 and 5.0, respectively. Therefore, the specifications do not suffer from multicollinearity problems.
